# An Efficient Synthesis of 1-(1,3-Dioxoisoindolin-2-yl)-3-aryl Urea Analogs as Anticancer and Antioxidant Agents: An Insight into Experimental and *In Silico* Studies

**DOI:** 10.3390/molecules29010067

**Published:** 2023-12-21

**Authors:** Obaid Afzal, Mohamed Jawed Ahsan

**Affiliations:** 1 Department of Pharmaceutical Chemistry, College of Pharmacy, Prince Sattam Bin Abdulaziz University, Al-Kharj 11942, Saudi Arabia; 2 Department of Pharmaceutical Chemistry, Faculty of Pharmacy, Jahangirabad Institute of Technology (JIT), Jahangirabad Fort, Jahangirabad 225203, Uttar Pradesh, India; jawedpharma@gmail.com

**Keywords:** ADMET, anticancer, antioxidant, cell lines, phthalimide, molecular docking

## Abstract

The present investigation reports the efficient multistep synthesis of 1-(1,3-dioxoisoindolin-2-yl)-3-aryl urea analogs (**7a**–**f**) in good yields. All the 1-(1,3-dioxoisoindolin-2-yl)-3-aryl urea analogs (**7a**–**f**) were characterized by spectroscopic techniques. Five among the six compounds were tested against 56 cancer cell lines at 10 µM as per the standard protocol. 1-(4-Bromophenyl)-3-(1,3-dioxoisoindolin-2-yl)urea (**7c**) exhibited moderate but significant anticancer activity against EKVX, CAKI-1, UACC-62, MCF7, LOX IMVI, and ACHN with percentage growth inhibitions (PGIs) of 75.46, 78.52, 80.81, 83.48, 84.52, and 89.61, respectively. Compound **7c** was found to exhibit better anticancer activity than thalidomide against non-small cell lung, CNS, melanoma, renal, prostate, and breast cancer cell lines. It was also found to exhibit superior anticancer activity against melanoma cancer compared to imatinib. Among the tested compounds, the 4-bromosubstitution (**7c**) on the phenyl ring demonstrated good anticancer activity. Docking scores ranging from −6.363 to −7.565 kcal/mol were observed in the docking studies against the molecular target EGFR. The ligand **7c** displayed an efficient binding against the EGFR with a docking score of −7.558 kcal/mol and displayed an H-bond interaction with Lys745 and the carbonyl functional group. Compound **7c** demonstrated a moderate inhibition of EGFR with an IC_50_ of 42.91 ± 0.80 nM, in comparison to erlotinib (IC_50_ = 26.85 ± 0.72 nM), the standard drug. The antioxidant potential was also calculated for the compounds (**7a**–**f**), which exhibited good to low activity. 1-(2-Methoxyphenyl)-3-(1,3-dioxoisoindolin-2-yl)urea (**7f**) and 1-(4-Methoxyphenyl)-3-(1,3-dioxoisoindolin-2-yl)urea (**7d**) demonstrated significant antioxidant activity with IC_50_ values of 15.99 ± 0.10 and 16.05 ± 0.15 µM, respectively. The 2- and 4-methoxysubstitutions on the *N*-phenyl ring showed good antioxidant activity among the series of compounds (**7a**–**f**). An in silico ADMET prediction studies showed the compounds’ adherence to Lipinski’s rule of five: they were free from toxicities, including mutagenicity, cytotoxicity, and immunotoxicity, but not for hepatotoxicity. The toxicity prediction demonstrated LD_50_ values between 1000 and 5000 mg/Kg, putting the compounds either in class IV or class V toxicity classes. Our findings might create opportunities for more advancements in cancer therapeutics.

## 1. Introduction

With nearly 10 million fatalities and nearly 19.3 million new cases of cancer worldwide in 2020, cancer is currently the leading cause of death. The number of cancer cases is expected to increase to 28.4 million by 2040 [1]. Cancer’s etiology is complex, involving the uncontrolled growth of cells invading neighboring tissues or organs. Adults over 50 are typically prone to developing cancer, but incidence rates of early-onset cancer (those diagnosed before 50) have increased globally [2,3]. In 2020, the global lifetime risk of cancer from birth to death was estimated to be 25.10% [4]. The United States is expected to have 1,958,310 new cancer cases and 609,820 cancer deaths in 2023 [5]. Similarly, the European Union (EU-27) is expected to have 1,261,990 cancer deaths in 2023, while the estimated number of cancer incidents in India in 2022 was 1,461,427 [6,7]. For many years, patients with cancer had only a limited number of treatment options, which included surgery, radiation therapy, and chemotherapy either individually or in combination [8]. Surgery offers the best chance of a cure for tumors that can be removed, especially in cases of early-stage disease [9]. Despite its known harmful effects on the patient’s physical as well as psychological health, chemotherapy is still a frequently chosen therapeutic option used either alone or in combination with radiotherapy [8,10]. One can avoid early-onset cancers by altering their diet, giving up alcohol, and quitting smoking [2]. Medicinal chemistry involves the invention, discovery, identification, synthesis of organic compounds with medicinal importance, in silico studies, target identification, and structure–activity relationship studies [11]. Medicinal chemists are crucial players in the process of discovering new, more effective drugs, and they are intensely engaged in cancer research worldwide to find a more pleasant cancer treatment option.

**Figure 1:**
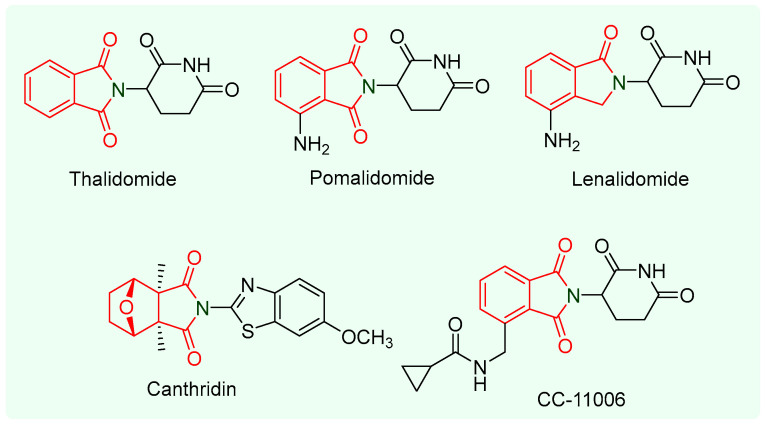
Phthalimide-based anticancer agents.

Heterocyclic compounds have drawn the attention of scientists due to their ever-increasing need for therapeutic compounds. Phthalimide is a fused heterocyclic ring with a variety of therapeutic uses, including antibacterial [12], antiviral [13], anticancer [14,15,16], anticonvulsant [17], anti-EGFR [15], anti-inflammatory [18], inflammatory and neuropathic pain [19], antimalarial [20], schistosomiasis [21], and many more [22,23]. Some of phthalimide-based anticancer compounds include cantharidin, CC-11006, lenalidomide, pomalidomide, and thalidomide (Figure 1) [24,25]. Inspired by the enormous therapeutic potential of phthalimide, we report herein the synthesis of a series of six phthalimide-based 1-(1,3-dioxoisoindolin-2-yl)-3-aryl urea analogs (**7a**–**f**) and their anticancer and antioxidant activities. Recent years have seen incredible progress in the expansion of anticancer drugs, and a number of molecular targets have been explored [26]. DNA topoisomerase I, epidermal growth factor receptor, focal-adhesion kinase, glycogen synthase kinase-3, histone deacetylase, methionine aminopeptidase, nuclear factor, poly(ADP-ribose) polymerase-1, tubulin, telomerase, thymidine phosphorylase, thymidylate synthase, and vascular endothelial growth factor receptor are some of the important targets reported in the literature [15,27]. 1-(1,3-Dioxoisoindolin-2-yl)-3-aryl urea analogs (**7a**–**f**) were subjected to molecular docking studies against three different molecular targets including DNA topoisomerase I, tubulin, and the EGFR. Phthalimide analogs were also reported as an inhibitor of the EGFR in previous studies; hence, we explored the binding affinity of compounds **7a**–**f** with the EGFR [15]. Many anticancer drugs, including gefitinib, laptinib, orantinib, nintedanib, semaxanibs, sunitinib, etc., have the EGFR as one of their primary molecular targets.

The body constantly produces free radicals, which can become toxic in high concentrations or when the body’s natural antioxidant defenses are not working properly [28]. High concentrations of free radicals cause lipids, proteins, and DNA damage in cells and tissues, which lead to serious illnesses like cancer, inflammatory diseases, CVS disorders, immunosuppression, and neurodegenerative disorders [29]. Cancer may develop as a result of free radicals’ formation brought on by oxidative stress. Antioxidants are a class of compounds that aid in the capture and neutralization of free radicals, reducing the harm that free radicals can cause to the body. In addition, antioxidants help prevent oxidative-stress-related illnesses like cancer and inflammation [30,31,32]. The antioxidant activity of 1-(1,3-dioxoisoindolin-2-yl)-3-aryl urea analogues (**7a**–**f**) was also assessed in the current study because phthalimide analogues have also been reported to be antioxidants [33,34,35]. Earlier a few 1-(1,3-dioxoisoindolin-2-yl)-3-aryl urea analogues were prepared and reported by researchers by two different routes. One research group prepared 1-(1,3-dioxoisoindolin-2-yl)-3-aryl urea analogues by refluxing substituted phenyl semicarbazide and phthalic anhydride in glacial acetic acid for 5 h with very low yields ranging from 41 to 61 percent [36]. Another group of researchers also prepared these compounds by refluxing *N*-aminophthalimide and *p*-substituted phenylcarbamate in ethanol for 1–2 h with yields varying from 35.9 to 86.2 percent [37]. We have opted for an alternative synthetic pathway for the synthesis, as it was discovered to be highly effective in terms of its yield. Earlier these compounds were reported as anticonvulsant and antiviral agents [36,37]. Since some of the compounds were reported to be non-toxic (cytotoxicity ≥ 400 µg/mL), it made sense to investigate their anticancer potential using cell line experiments [37].

Modern therapeutic development is aided by in silico studies. During the COVID-19 pandemic, bioinformatics and in silico techniques were valued [38]. In silico screening methods based on algorithms rapidly screen numerous molecules to identify potential drugs [39]. Thanks to cutting-edge in silico and artificial intelligence techniques, research is no longer limited to laboratory settings. This has resulted in the development of INS018_055, a first-in-class AI-generated anti-fibrotic medication that is undergoing a multiregional Phase II clinical trial in both China and the United States [40,41,42]. The current study aimed to synthesize a few 1-(1,3-dioxoisoindolin-2-yl)-3-aryl urea analogues (**7a**–**f**), evaluate their biological potentials, and investigate in silico docking and ADMET prediction.

## 2. Results

### 2.1. Chemistry

**Scheme 1:**
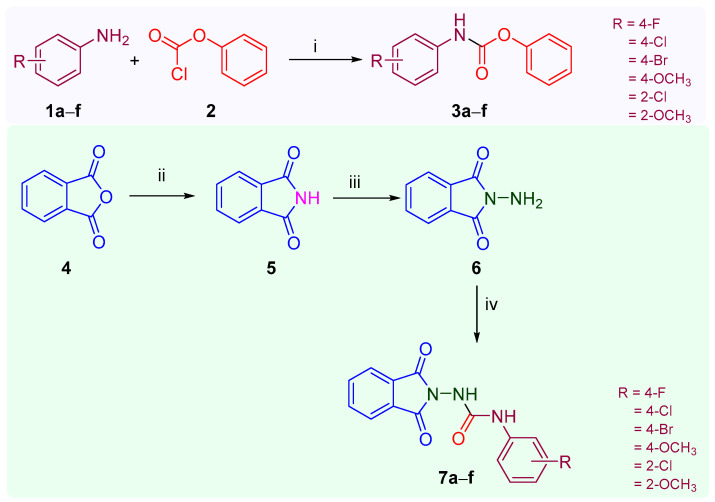
Synthetic protocol of 1-(1,3-dioxoisoindolin-2-yl)-3-(substituted phenyl)urea analogs (**7a**–**f**): Reagents and conditions: (i) CHCl_3_, Et_2_N, rt, 4–5 h, 78–80% yield%; (ii) NH_2_CONH_2_, 140 °C, 5 min, 86%, yield; (iii) EtOH, NH_2_NH_2_.H_2_O; 85 °C, 5 min, 72 yield; (iv) **3a**–**f**, DCM, rt, 6–8 h, 81–92% yield.

The protocol for the synthesis of 1-(1,3-dioxoisoindolin-2-yl)-3-aryl urea analogs (**7a**–**f**) is summarized in Scheme 1. In the initial step, phenyl(substituted phenyl)carbamate (**3a**–**f**) was prepared by adding a solution of substituted aniline (**1a**–**f**) (2.5 mmol) in triethylamine to a solution of phenyl chloroformate (**2**) in chloroform [34]. In the second step, phthalimide (**5**) was prepared through the fusion of phthalic anhydride (**4**) and urea at 140 °C, and in the subsequent step, phthalimide (**5**) was allowed to react with hydrazine hydrate (NH_2_NH_2_.H_2_O) in ethanol at 85 °C for 5 min to obtain 2-aminoisoindoline-1,3-dione (**6**) [43]. In the concluding step, an equimolar quantity of phenyl(substituted phenyl)carbamate (**3a**–**f**) and aminoisoindoline-1,3-dione (**6**) in dichloromethane (DCM) was continuously stirred for 6–8 h at room temperature to obtain the final product, 1-(1,3-dioxoisoindolin-2-yl)-3-aryl urea analogs (**7a**–**f**) [43]. Preparatory thin-layer chromatography (TLC Silica gel 60 F_254_) using toluene/ethylacetate/formic acid (5:4:1) as the mobile phase was used to continuously track the reaction’s progress. The compounds (**7a**–**f**) were identified and characterized based on the spectral data as well as their melting points reported in the previous literature. Yogeeswari et al. reported the synthesis of 1-(1,3-dioxoisoindolin-2-yl)-3-aryl urea analogs from phthalic anhydride and substituted phenyl semicarbazide in glacial acetic acid with very low yields ranging from 41 to 61 percent. Similarly, Verma et al. reported their synthesis with low to good yields ranging from 35.9 to 86.2 percent [36,37]. The title compounds (**7a**–**f**) were successfully synthesized with excellent yields ranging from 81 to 92 percent in the current investigation using a productive synthetic scheme (Scheme 1). The physical constant and NSC code of compounds (**7a**–**f**) are given in Table 1. The title compounds (**7a**–**f**) were identified using spectroscopic techniques, such as mass and NMR spectral data. The ^1^H NMR of the title compounds (**7a**–**f**) showed a singlet at δ ppm 3.74 corresponding to the three protons of the OCH_3_ group present in the compounds **7d** and **7f**; a multiplet at δ ppm 7.88–8.15 corresponding to the four aromatic protons of phthalimide; two multiplets at δ ppm 7.33–7.42 (H3 and H5) and 7.48–7.60 (H2 and H6) corresponding to each of the two protons of the 4-flurophenyl ring of compound **7a**; two doublets at δ ppm ranging from 6.95 to 7.43 (H3 and H5) and 7.31 to 7.74 (H2 and H6) corresponding to each of the two aromatic protons of the substituted phenyl ring of compound **7b**–**d**; and two multiplets at δ ppm 7.05–7.52 and 7.53–7.98 corresponding to each of four the aromatic protons of the *N*-phenyl ring and phenyl ring of phthalimide, respectively, for the compounds **7e** and **7f**. Similarly, a singlet ranging from δ ppm 9.59 to 9.69 corresponding to the secondary amine (ArNH) was observed, while a singlet ranging from δ ppm 10.03 to 10.65 was observed, corresponding to the amide (CONH) group present in the compounds (**7a**–**f**). The ^13^C NMR spectroscopy identified the nature and number of carbons present in any of the compounds (**7a**–**f**). The mass spectra (ESI-MS) of the compounds (**7a**–**f**) revealed (M + 1)^+^ (**7a**–**f**) and (M + 3)^+^ (**7b**,**c**, and **7e**) peaks corresponding to their molecular formula. One of the representative compounds, **7c**, had a purity of 98.74 percent in HPLC.

### 2.2. Anticancer Activity

**Figure 2:**
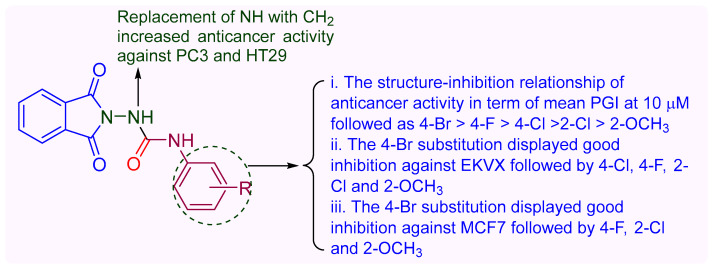
The structure–inhibition relationship for anticancer activity of 1-(1,3-dioxoisoindolin-2-yl)-3-aryl urea analogs.

According to the reported protocol, the anticancer activity of the compounds was evaluated against the nine diverse panels of 56 NCI cancer cell lines at 10 µM in terms of their growth control [44,45,46,47]. When tested against the breast cancer cell line MCF7, compound **7e** showed anticancer activity with a growth percentage (GP) of 87.43%, while compound **7f** showed anticancer activity when tested against the renal cancer cell line CAKI-1 with a GP of 89.58%. Compound **7a** exhibited anticancer activity against the MCF7 (breast cancer), HL-60(TB) (leukemia), and EKVX (non-small cell lung cancer) cancer cell lines with GPs of 84.87, 86.23, and 89.44 percent, respectively. Compound 7**b** exhibited moderate anticancer activity against the EKVX (non-small cell lung cancer), CAKI-1 (renal cancer), HL-60(TB) (leukemia), UACC-62 (melanoma), and LOX IMVI (melanoma) cancer cell lines with GPs of 80.73, 82.3, 83.07, 85.6, and 87.19 percent, respectively. Compound **7c** exhibited significant anticancer activity against the EKVX (non-small cell lung cancer), CAKI-1 (renal cancer), UACC-62 (renal cancer), MCF7 (breast cancer), LOX IMVI (melanoma), and ACHN (renal cancer) cancer cell lines with GPs of 75.46, 78.52, 80.81, 83.48, 84.52, and 89.61, respectively, among the series of compounds. The anticancer data of the compounds at 10 µM are given in Table 2. The five most sensitive cell lines are summarized in Table 3. The compounds’ average percentage growth inhibitions (PGIs) against each panel were calculated and compared to the reference drug, imatinib (Table 4). The compounds **7a**–**c** and **7e**,**f** demonstrated good inhibition against melanoma and ovarian cancer compared to imatinib. Compound **7c** displayed better anticancer activity than imatinib (PGI = −0.87%) against melanoma with a percentage growth inhibition (PGI) of 5.03 percent. The compounds **7a**–**c** and **7e**,**f** demonstrated good inhibition against CNS, melanoma, and prostate cancer compared to thalidomide. Compound **7c** displayed better anticancer activity than thalidomide against the non-small cell lung, CNS, melanoma, renal, prostate, and breast cancer cell lines. The anticancer activity of the compounds **7a**–**c** and **7e**,**f** was found to be less than that of gefitinib. The 4-bromo substitution on the *N*-phenyl ring demonstrated promising results. The structure–activity relationship using the mean PGI was measured for the following substitutions: *N*-phenyl 4-bromo > 4-fluoro > 4-chloro > 2-chloro > 2-methoxy. Similarly, the structure–activity relationship against the EKVX and MCF7 cell lines are shown in Figure 2. The replacement of the NH function with CH_2_ increased the anticancer activity against PC3 and HT29 as evidenced from previous reported work [48]. Previous reports have demonstrated a decrease in anticancer activity against PC3 and HT29 when the NH function was replaced with CH_2_ together with the replacement of the phenyl ring with the bulky benzylthio-1,3,4-thiadiazole moiety [49]. Further research is required to determine whether the substitution of the 1,3-dioxoisoindole moiety affects the anticancer activity, as in some cases, the substitution of the 1,3-dioxoisoindole moiety alters the anticancer activity [24,25]. Other changes, such as replacing NH with CH_2_ or C=O with C=S, may be possible and could be the focus of future studies on SAR establishment. The anticancer data of the compounds against 56 NCI cancer cell lines can be found within the Supplementary Material (Figures S1–S5).

### 2.3. In Vitro EGFR Kinase Inhibition Assay

In order to investigate the EGFR inhibition potential of the most active anticancer compound (**7c**), an EFGR kinase assay kit (BPS Bioscience, San Diego, CA, USA) was used as per the manufacturer’s instructions and the protocol is reported [50]. The IC_50_ values for the tested compound (**7c**) and the reference drug (erlotinib) were determined in a molar concentration using the GraphPad prism 9 software. The inhibition assays were performed in triplicate and their results showed that the compound displayed an inhibition of the EGFR with an IC_50_ value of 42.91 ± 0.80 nM, while the standard drug erlotinib displayed an IC_50_ value of 26.85 ± 0.72 nM. Compound **7c** displayed less EGFR inhibitory activity than erlotinib. 

### 2.4. Molecular Docking Studies

**Figure 3:**
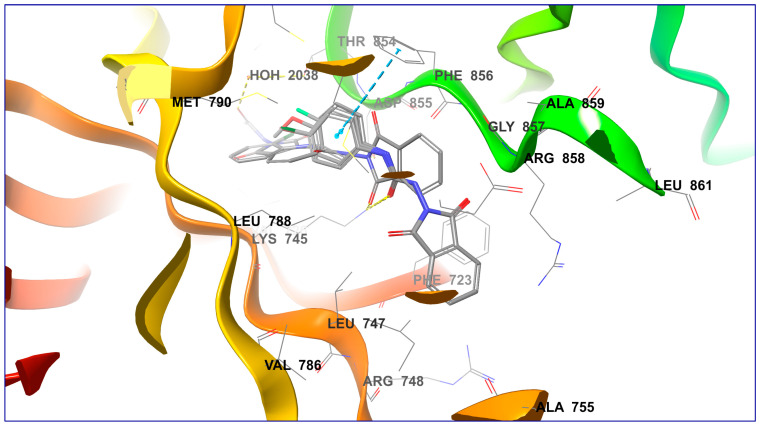
The 3D interaction of ligands (**7a**–**f**) with EGFR (PBD: 3W2R).

**Figure 4:**
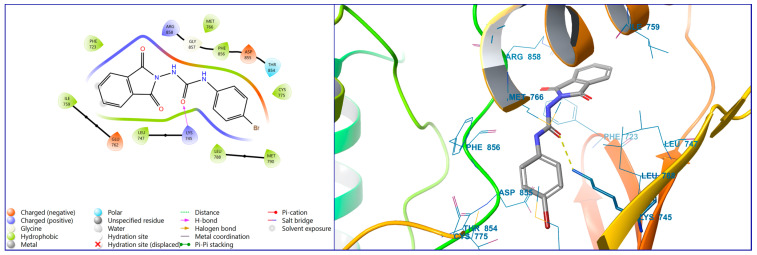
The 2D and 3D interactions of ligand **7c** with EGFR (PBD: 3W2R).

**Figure 5:**
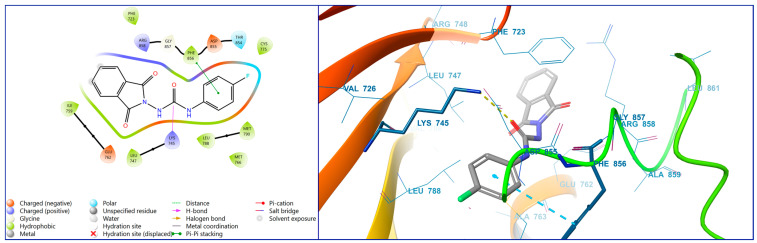
The 2D and 3D interactions of ligand **7a** with EGFR (PBD: 3W2R).

The molecular docking of the ligands (**7a**–**f**) was studied against the three important molecular targets of anticancer drugs: tubulin, DNA topoisomerase I, and the epidermal growth factor receptor (EGFR). Higher eukaryotes depend on DNA topoisomerase I enzymes to relax DNA supercoils caused by transcription, replication, and chromatin remodeling [51]. It is the target of the commonly used anticancer medications topotecan and irinotecan, which are made from the alkaloid camptothecin [52,53]. In vitro and in vivo studies of a fluoroglycosyl-3,9-difluoroindolocarbazole (BMS-250749) containing the phthalimide moiety demonstrated DNA topoisomerase I’s inhibitory activity; therefore, we selected DNA topoisomerase I for our molecular docking studies [54,55]. Microtubules are an important target for cancer therapy because they are essential for many cellular processes, such as mitosis, cell signaling, and organelle trafficking [56]. The development of novel tubulin inhibitors (TIs) that specifically target the colchicine binding site seems to be a promising avenue for tubulin inhibitor advancement [57]. Paclitaxel, docetaxel, epothilones, vincristine, vinblastine, vinflunine, dolastatin 10, colchicine, podophyllotoxin, and noscapine are some of the reported tubulin inhibitors [58]. Phthalimide analogues were also reported as a tubulin inhibitor [59]. From the protein data bank, the X-ray crystallographic structure of DNA topoisomerase I (PDB ID: 1SC7) and tubulin (PDB ID: 1SA0) was retrieved for docking studies [60,61]. Docking scores ranging from −5.830 to −7.292 kcal/mol were observed in the docking studies of ligands **7a**–**f** against tubulin as a molecular target (PDB ID: 1SA0). The binding site of colchicine in tubulin enzyme (PDB ID: 1SA0) consists of a hydrophobic cavity, lined with a few active residues, like Lys254, Cys241, Asn248, Lys352, Ala316, Val315, Met259, Leu248, Ala250, and Ala317. It contains glycine 717, the polar residues Thr718 and Asn722, the hydrophobic residue Leu721, and the positively charged residue Arg364. Similarly, docking scores of ranging from −6.363 to −7.565 kcal/mol were observed in the docking studies of ligands **7a**–**f** against human DNA topoisomerase I (PDB ID: 1SC7). An efficient binding was observed with the EGFR as a putative target with docking scores ranging from −7.702 to −8.644 kcal/mol. The active site of DNA topoisomerase I (PDB ID: 1SC7) contains glycine 717, the polar residues Thr718 and Asn722, the hydrophobic residue Leu721, and the positively charged residue Arg364. The EGFR is an important molecular target for many anticancer drugs, including gefitinib, laptinib, orantinib, nintedanib, semaxanibs, sunitinib, etc. [62]. From the protein data bank, the X-ray crystallographic structure of the EGFR (PDB ID: 3W2R) was retrieved for the docking studies [63]. The ligands **7a**–**f** were saved as mol files and were prepared for docking using Ligprep, and the docking was carried out in accordance with the method outlined by Sogabe et al. (2013) [64]. The active site contains the hydrophobic residues Phe723, Leu747, Ile759, Met766, Cys775, Leu788, Met790, and Phe856; the positively charged residues Lys745 and Arg858; the negatively charged residues Glu762 and Asp855; and the polar residue Thr854. The docking studies with two types of electrostatic interactions, H-bond and π–π stacking, showed docking scores ranging from −6.363 to −7.565 kcal/mol. The ligands **7d** and **7c** showed a similar type of binding interactions with the residue Lys745 through an H-bond, whereas the ligands **7a** and **7d** showed identical interactions with the residue Lys745 through an H-bond and π–π stacking with the residue Phe856. The ligand **7e** showed two H-bond interactions with the residues Lys745 and Asp855, while the ligand **7f** displayed an H-bond interaction with Thr854 via the water molecule. The results of the molecular docking studies are compiled in Table 5, and Figure 3 displays the interaction of the ligands three-dimensionally (**7a**–**f**). The two- and three-dimensional interactions of ligand **7c** are shown in Figure 4, while the two- and three-dimensional interactions of ligand **7a** are shown in Figure 5. The electrostatic interaction in the molecular docking studies of 1-(1,3-dioxoisoindolin-2-yl)-3-aryl urea analogs (**7a−f**) against tubulin (PDB ID: 1SA0) and DNA topoisomerase I (PDB ID: 1SC7) are given in Table S1 (Supplementary Materials).

### 2.5. Antioxidant Activity

**Figure 6:**
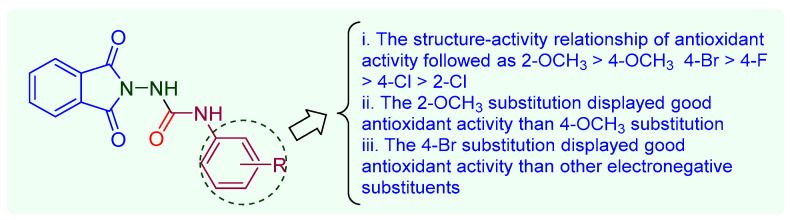
The structure–activity relationship for antioxidant activity of 1-(1,3-dioxoisoindolin-2-yl)-3-aryl urea analogs.

Oxidative stress causes inflammation and transforms normal cells into cancerous ones [65,66,67]. Free radical formation as a result of oxidative stress plays an important role in the progress of many chronic illnesses, including CVS disorders, ageing, anaemia, cancer, and inflammation. Antioxidants provide protection against these disease by scavenging available free radicals [68,69]. The antioxidant activity was calculated as the IC_50_ in µM, using the DPPH free radicals scavenging activity as described by Koleva et al., 2002, and is given in Table 6 [70]. The compounds displayed antioxidant activity with IC_50_ values ranging between 15.99 ± 0.10 and 54.27 ± 0.49 µM. The compounds **7f** and **7d** demonstrated significant antioxidant activity with IC_50_ values of 15.99 ± 0.10 and 16.05 ± 0.15 µM, respectively, while rest of the compounds displayed moderate to low antioxidant activity in comparison to the standard ascorbic acid. The compound **7c** demonstrated moderate antioxidant activity with IC_50_ values of 24.02 ± 0.29. The 2- and 4-methoxy substitution on the 3-aryl ring displayed promising results, while rest of the compounds containing electronegative group substitutions (4-fluoro, 4-chloro, 4-bromo, and 2-chloro) at the 2 or 4 positions displayed moderate to low antioxidant activity (Figure 6). As demonstrated in earlier, adding an ethylene linkage between an amide and an aryl NH enhanced the antioxidant potential for the 4-methoxy substitution on the phenyl ring [71]. Additionally, studies are needed to ascertain whether the substitution of the 1,3-dioxoisoindole moiety influences the antioxidant activity. Other modifications, such as replacing NH with CH_2_ or C=O with C=S, may be feasible and will be the focus of future research to establish SAR.

### 2.6. ADMET Prediction

ADMET predictions are indispensable in drug discovery programs to reduce the possibility of pharmacokinetics-related clinical failures [72]. Pharmaceutical companies suffer significant financial losses as a result of ADMET-related post-marketing failures of drug candidates [73]. ADMET properties are crucial variables that must be researched during clinical trials for drug development. Since evaluating every compound in in vivo ADMET studies is a laborious and time-consuming process, in silico studies could be of significant help in reducing such clinical failures. All the compounds were subjected to ADMET predictions using the software programs swissADME and ProTox II, which are available for free [74,75]. The compounds (**7a**–**f**) demonstrated that they adhered to Lipinski’s rule of five and had molecular weights between 299 and 360.16 (MW < 500), log *p* values between 1.74 and 2.05 (log P ≤ 5), and a range of three to four hydrogen bond acceptors (HBAs) (<HBA 10) and two hydrogen bond donors (HBD < 5), among other characteristics [76]. The percent absorption (% ABS), which was calculated as $\%\;Abs=109\pm \left[0.345\times TPSA\right]$, was found to be 78.73 and 81.91 percent [77]. The in silico ADMET prediction results are summarized in Table 7. The compounds were also predicted to be toxicity-free, with the exception of hepatotoxicity (HEP). The toxicity prediction demonstrated LD_50_ values between 1000 and 5000 mg/Kg, putting the compounds in class IV (300 < LD_50_ ≤ 2000) and class V (2000 < LD_50_ ≤ 5000) toxicity classes [75]. The in silico toxicity prediction results are summarized in Table 8. Some of the compounds (**7a**–**d**) were reported to be non-toxic (cytotoxicity > 400 µg/mL) in a previous report [37].

## 3. Discussion

Phthalimide analogs have been reported to be biologically active compounds, and a number of biological activities have been reported to date. Inspired by their biological activities we report herein the synthesis of as well as anticancer, antioxidant, and in silico studies on 1-(1,3-dioxoisoindolin-2-yl)-3-aryl urea analogs (**7a**–**f**). The synthesis of the compounds (**7a**–**f**) were accomplished in multiple steps as per the reported protocols [36,37,43]. Yogeeswari et al. synthesized 1-(1,3-dioxoisoindolin-2-yl)-3-aryl urea analogs from phthalic anhydride and substituted phenyl semicarbazide in glacial acetic acid with very low yields ranging from 41 to 61 percent. Similarly, Verma et al. synthesized (1,3-dioxoisoindolin-2-yl)-3-aryl urea analogs with low to good yields ranging from 35.9 to 86.2 percent [36,37]. Phenyl(substituted phenyl)carbamate (**3a**–**f**) was prepared from substituted aniline (**1a**–**f**) and phenyl chloroformate (**2**) in the first step, while aminoisoindoline-1,3-dione (**6**) was prepared in two steps starting from phthalic anhydride (**4**). In the final step, aminoisoindoline-1,3-dione (**6**) and phenyl(substituted phenyl)carbamate (**3a**–**f**) was stirred in DCM at room temperature for 6–8 h to obtain 1-(1,3-dioxoisoindolin-2-yl)-3-aryl urea analogs (**7a**–**f**); however, in a previous work, 1-(1,3-dioxoisoindolin-2-yl)-3-aryl urea analogs were prepared from aminoisoindoline-1,3-dione and ethyl *N*-*p*-substituted phenyl carbamate but the yield was low, with some exceptions (a few compounds were obtained in good yields) [37]. Overall, the synthetic protocol reported herein was found to be efficient with good yields. Five compounds were assessed for their anticancer activity against 56 cancer cell lines in single dose assay at 10 µM. Among the series, compound **7c** demonstrated a moderate but significant anticancer activity against EKVX, CAKI-1, UACC-62, MCF7, LOX IMVI, and ACHN with GP of 75.46, 78.52, 80.81, 83.48, 84.52, and 89.61, respectively. Compound **7c** displayed better anticancer activity than imatinib against melanoma with a PGI of 5.03 percent. The EGFR is an important target for many anticancer drugs; hence, the compounds were subjected to docking studies against the EGFR. Efficient binding with two types of electrostatic interactions, H-bond and π–π stacking interactions, and docking scores ranging from −6.363 to −7.565 kcal/mol was observed in the docking studies. The ligand **7c** displayed an H-bond interaction with Lys745 with a docking score of −7.558 kcal/mol. Free radical formation as a result of oxidative stress could be another reason to develop cancer; hence, the in vitro antioxidant potentials of the compounds **7a**–**f** was also studied. The compounds displayed an antioxidant activity with IC_50_ values ranging between 15.99 ± 0.10 and 54.27 ± 0.49 µM. The compounds **7f** and **7d** demonstrated significant antioxidant activity with IC_50_ values of 15.99 ± 0.10 and 16.05 ± 0.15 µM, respectively, while rest of the compounds displayed moderate to low antioxidant activity in comparison to the standard ascorbic acid. The in silico ADMET prediction displayed the compounds’ adherence to Lipinski’s rule of five and they were predicted to be free from toxicities, except for hepatotoxicity (HEP). The toxicity prediction demonstrated LD_50_ values between 1000 and 5000 mg/Kg, putting the compounds either in class IV (300 < LD_50_ ≤ 2000) or class V (2000 < LD_50_ ≤ 5000) toxicity classes. Overall, in the present investigation, an efficient synthesis of 1-(1,3-dioxoisoindolin-2-yl)-3-aryl urea analogs (**7a**–**f**) was accomplished adopting different synthetic routes, followed by studies on their biological activities and in silico ADMET predictions.

## 4. Materials and Methods

### 4.1. General Procedure for the Synthesis of Phenyl(substituted phenyl)carbamate Analogs *(**3a**–**f**)*

A solution of 2- and 4-substituted aniline (**1a**–**f**) (1 mmol) in triethylamine (1 mmol; ~2.8 mL) was added dropwise to a solution of phenyl chloroformate (1 mmol; 1.30 mL) in chloroform (5 mL), and reaction mixture was continuously stirred with magnetic stirrer at room temperature (rt) for 4–5 h. The reaction mixture was then concentrated under vacuum and cooled to room temperature. Petroleum ether (20 mL) was added to obtain precipitate, which was filtered, washed with water, and dried at room temperature to obtain phenyl (substituted phenyl)carbamate (**3a**–**f**).

### 4.2. Procedure for the Synthesis of Phthalimide *(**5**)*

Equimolar amounts of phthalic anhydride (25 mmol; 3.70 g) (**4**) and urea (25 mmol; 1.5 g) were fused at high temperature (140 °C) for 5 min, then the reaction mixture was cooled to obtain white precipitate. The solid was triturated with hydroalcoholic (H_2_O-EtOH, 2:1) solution to obtain phthalimide (**5**).

### 4.3. Procedure for the Synthesis of 2-Aminoisoindoline-1,3-dione *(**6**)*

Equimolar amounts of phthalimide (**5**) (20 mmol; 2.94 g) and hydrazine hydrate (20 mmol; 0.65 mL) in absolute ethanol (50 mL) were shaken at room temperature for 2 min followed by quickly heating them for 3 min in water bath. The reaction mixture was then poured into crushed ice to obtain a precipitate of 2-aminoisoindoline-1,3-dione (**6**).

### 4.4. General Procedure for the Synthesis of 1-(1,3-Dioxoisoindolin-2-yl)-3-aryl Urea Analogs *(**7a**–**f**)*

An equimolar mixture of phenyl(substituted phenyl)carbamate (**3a**–**f**) (1 mmol) and aminoisoindoline-1,3-dione (**6**) (1 mmol; 162 mg) in dichloromethane (DCM) (10 mL) was continuously stirred for 6–8 h at room temperature. The precipitate of the final 1-(1,3-dioxoisoindolin-2-yl)-3-aryl urea analogs (**7a**–**f**) was separated out, filtered, washed with DCM, and dried at room temperature, followed by recrystallization from ethanol.

*1-(4-Fluorophenyl)-3-(1,3-dioxoisoindolin-2-yl)urea* (**7a**): ^1^H NMR (300 MHz, DMSO-*d*_6_): δ ppm: 7.35–7.42 (m, 2H, ArH), 7.48–7.60 (m, 2H, ArH), 7.88–8.15 (m, 4H, ArH), 9.69 (s, 1H, ArNH), 10.65 (s, 1H, CONH); ^13^C NMR (75 MHz, DMSO-*d*_6_): δ ppm: 167.2, 163.1, 154.4, 135.1, 132.5, 132.1, 124.1, 119.9, 116.2; Anal. Calc. for C_15_H_10_FN_3_O_3_: C, 60.20; H, 3.37; N, 14.04 found: C, 60.01; H, 3.35; N, 14.05%. ESI-MS *m*/*z* = 300 (M + 1)^+^, 301 (M + 2)^+^.

*1-(4-Chlorophenyl)-3-(1,3-dioxoisoindolin-2-yl)urea* (**7b**): ^1^H NMR (300 MHz, DMSO-*d*_6_): δ ppm: 7.33 (d, 1H, *J* = 6.9 Hz, ArH), 7.49 (d, 1H, *J* = 9.0 Hz, ArH), 7.61 (d, 2H, *J* = 6.1 Hz, ArH), 7.88–8.15 (m, 4H, ArH), 9.69 (s, 1H, ArNH), 10.03 (s, 1H, CONH); ^13^C NMR (75 MHz, DMSO-*d*_6_): δ ppm: 166.2, 154.9, 137.5, 133.9, 132.6, 132.1, 129.3, 123.9, 120.9; Anal. Calc. for C_15_H_10_ClN_3_O_3_: C, 57.07; H, 3.19; N, 13.31 found: C, 57.00; H, 3.20; N, 13.35%. ESI-MS *m*/*z* = 316 (M + 1)^+^, 318 (M + 3)^+^.

*1-(4-Bromophenyl)-3-(1,3-dioxoisoindolin-2-yl)urea* (**7c**): ^1^H NMR (300 MHz, DMSO-*d*_6_): δ ppm: 7.43 (d, *J* = 8.7 Hz, 2H, ArH), 7.74 (d, *J* = 8.4 Hz, 2H, ArH), 7.89–8.14 (m, 4H, ArH), 9.62 (s, 1H, ArNH), 10.09 (s, 1H, CONH); ^13^C NMR (75 MHz, DMSO-*d*_6_): δ ppm: 164.9, 154.7, 139.1, 132.9, 132.1, 131.9, 123.9, 122.7, 121.9; Anal. Calc. for C_15_H_10_BrN_3_O_3_: C, 50.02; H, 2.80; N, 11.67 found: C, 49.98; H, 2.78; N, 11.70%. ESI-MS *m*/*z* = 360 (M + 1)^+^, 362 (M + 3)^+^. HPLC purity: 98.74%.

*1-(1,3-Dioxoisoindolin-2-yl)-3-(4-methoxyphenyl)urea* (**7d**): ^1^H NMR (300 MHz, DMSO-*d*_6_): δ ppm: 3.74 (s, 3H, OCH_3_), 6.95 (d, *J* = 6.9 Hz, 2H, ArH), 7.31 (d, *J* = 6.6 Hz, 2H, ArH), 7.88–8.02 (m, 4H, ArH); 9.59 (s, 1H, ArNH), 10.53 (s, 1H, CONH); ^13^C NMR (75 MHz, DMSO-*d*_6_): δ ppm: 164.9, 159.1, 154.5, 139.9, 132.1, 131.9, 124.1, 120.1, 114.9, 56.2; Anal. Calc. for C_16_H_13_N_3_O_4_: C, 61.73; H, 4.21; N, 13.50 found: C, 61.69; H, 4.19; N, 13.53%. ESI-MS *m*/*z* = 312 (M + 1)^+^.

*1-(2-Chlorophenyl)-3-(1,3-dioxoisoindolin-2-yl)urea* (**7e**): ^1^H NMR (300 MHz, DMSO-*d*_6_): δ ppm: 7.53–7.72 (m, 3H, ArH), 7.93–7.98 (m, 1H, ArH), 8.00–8.15 (m, 4H, ArH), 9.69 (s, 1H, ArNH), 10.03 (s, 1H, CONH); ^13^C NMR (75 MHz, DMSO-*d*_6_): δ ppm: 164.9, 154.3, 134.9, 132.7, 132.1, 131.4, 130.5, 129.3, 125.3, 124.6, 123.8; Anal. Calc. for C_15_H_10_ClN_3_O_3_: C, 57.07; H, 3.19; N, 13.31 found: C, 57.02; H, 3.21; N, 13.34%. ESI-MS *m*/*z* = 316 (M + 1)^+^, 318 (M + 3)^+^.

*1-(1,3-Dioxoisoindolin-2-yl)-3-(2-methoxyphenyl)urea* (**7f**): ^1^H NMR (300 MHz, DMSO-*d*_6_): δ ppm: 3.74 (s, 3H, OCH_3_), 7.05–7.52 (m, 4H, ArH), 7.82–8.07 (m, 4H, ArH); 9.63 (s, 1H, ArNH), 10.61 (s, 1H, CONH); ^13^C NMR (75 MHz, DMSO-*d*_6_): δ ppm: 164.9, 153.9, 149.7, 132.9, 132.1, 128.7, 124.8, 123.9, 121.6, 120.5, 112.9, 56.2; Anal. Calc. for C_16_H_13_N_3_O_4_: C, 61.73; H, 4.21; N, 13.50 found: C, 61.68; H, 4.19; N, 13.54%. ESI-MS *m*/*z* = 312 (M + 1)^+^.

### 4.5. Anticancer Activity

According to the reported protocol, 56 cancer cell lines were taken to test the compounds’ anticancer in one-dose assay at 10 µM [44,45,46,47]. The previous report contains a comprehensive procedure for anticancer screening [78]. 

### 4.6. In Vitro EGFR Kinase Inhibition Assay

In order to investigate the EGFR inhibition potential of most active anticancer compound (**7c**), EFGR kinase assay kit (BPS Bioscience, San Diego, CA, USA) was used as per the manufacturer’s instructions and the reported protocol [50]. To each well, the master mixture (25 µL) containing 5× Kinase Buffer 1 (6 µL), ATP (1 µL; 500 µM), PTK substrate Poly (Glu:Tyr 4:1) (1 μL; 10 mg/mL), and water (17 μL) was added. After pipetting master mixture, 5 µL solutions (in aqueous DMSO) of varying concentrations of **7c** and erlotinib, were added to the test inhibitor (**7c** and Erlotinib) wells. A total of 5 μL of inhibitor’s buffer (containing no inhibitors) was added to positive control and blank wells. Finally, 20 μL EGFR (1 ng/μL) was added to the positive control and test inhibitor wells, while 20 μL of 1× kinase buffer was added to the blank wells. Each well then contained a total of 50 μL solution and was incubated at 30 °C for 40 min. A total of 50 μL of Kinase-Glo MAX detection reagent (Promega, Madison, WI, USA) was then added and the wells were further incubated at 30 °C for 15 min under light protection. Luminescence intensity was measured using ELISA reader at 450 nm. IC_50_ values for the tested compound **7c** and the reference drug (erlotinib) were determined in µM using GraphPad prism 9 software at five different concentrations. The inhibition assays were performed in triplicate.

### 4.7. Molecular Docking Studies

From the protein data bank, the X-ray crystallographic structure with a resolution of 2.05 Å and R-value of 0.220 (observed) of the EGFR with PDB ID: 3w2r was retrieved for docking studies [63]. The ligands **7a**–**f** saved as mol files were prepared for docking using Ligprep, and protein was minimized. Afterwards, the grid was prepared and ligand docking was carried out as per the docking protocol reported by Sogabe et al., 2013 [64]. From the protein data bank, the X-ray crystallographic structure with a resolution of 3.00 Å and R-value of 0.233 of DNA topoisomerase I with PDB ID: 1sc7 was retrieved for docking studies, and ligand docking was carried out as per the docking protocol in the reported methods of [27,60]. From the protein data bank, the X-ray crystallographic structure with a resolution of 3.58 Å and R-value of 0.233 (observed) of the tubulin with PDB ID: 1as0 was retrieved for docking studies. Ligand docking was carried out as per the docking protocol reported in the methods of [59,79].

### 4.8. Antioxidant Activity

The compounds (**7a**–**f**) were tested for their antioxidant potentials using the DPPH free radical scavenging activity as described by Koleva et al., 2002 [68]. The IC_50_ is the concentration of test compounds (in µM) that showed 50% scavenging activity of free radicals, and this value was used to calculate the antioxidant activity. 

### 4.9. ADMET Prediction

All the compounds were subjected to ADMET prediction using the software programs swissADME and ProTox II, which are available for free [74,75].

## 5. Conclusions

The efficient multistep synthesis of 1-(1,3-dioxoisoindolin-2-yl)-3-aryl urea analogs (**7a**–**f**) was reported herein. Their anticancer activity against some cancer cell lines was found to be significant. Against a few cancer cell lines, including EKVX, CAKI-1, UACC-62, MCF7, LOX IMVI, and ACHN, compound 7**c** showed notable anticancer activity. In order to assess the compounds’ affinity towards the active site of the EGFR for potential inhibition, molecular docking studies were also conducted against the putative target EGFR. As the formation of free radicals as a result of oxidative stress may be another factor in the transformation of normal cells to cancerous ones, the antioxidant potentials of compounds **7a**–**f** were also investigated. Only the compounds **7f** and **7d** demonstrated notable antioxidant activity when compared to the antioxidant activity of ascorbic acid. Our in silico ADMET predictions showed the compounds’ adherence to the Lipinski’s rule of five and they were free from toxicities, except for hepatotoxicity. The toxicity prediction demonstrated LD_50_ values between 1000 and 5000 mg/Kg, putting the compounds either in class IV or class V toxicity classes. The present investigation may potentiate future drug discovery programs for anticancer drugs.

## Figures and Tables

**Table 1:** The comparative yields, melting point, and NSC code of 1-(1,3-dioxoisoindolin-2-yl)-3-aryl urea analogs (**7a**–**f**).

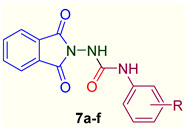								
S. No.	Compound	R	NSC Code	R_f_ *	% Yield		Mp (°C)	
					Found	Reported	Found	Reported
1	**7a**	4-Fluoro	838925	0.69	89	67.7 ^a^	248–250	>245 ^a^
2	**7b**	4-Chloro	838926	0.71	90	86.2 ^a^	252–254	>250 ^a^
3	**7c**	4-Bromo	838927	0.72	92	73.1 ^a^	250–252	250 ^a^
4	**7d**	4-Methoxy	-	0.67	83	35.9 ^a^	>260	>260 ^a^
5	**7e**	2-Chloro	838928	0.68	81	46 ^b^	140–142	139 ^b^
6	**7f**	2-Methoxy	838934	0.73	85	-	156–158	-

* toluene: ethyl acetate: formic acid (TEF, 5:4:1); ^a^ Verma et al. [37]; ^b^ Yogeeswari et al. [36].

**Table 2:** The anticancer activity of 1-(1,3-dioxoisoindolin-2-yl)-3-aryl urea analogs (**7a**–**f**) at 10 µM as GP.

Panel	Cell Line	7a	7b	7c	7e	7f
*Leukemia*	CCRF-CEM	103.9	104.8	101.2	103.37	95.13
	HL-60(TB)	86.23	83.07	92.99	101.97	101.22
	K-562	100.3	91.98	92.8	110.7	92.54
	MOLT-4	98.29	101.1	99.67	97.49	106.2
	RPMI-8226	105.9	106.5	108.3	109.46	103.93
	SR	94.86	96.2	94.2	92.66	93.35
*Non-small cell lung cancer*	A549/ATCC	99.08	102.7	101.2	103.02	103.59
	EKVX	89.44	80.73	75.46	90.83	91.2
	HOP-62	107.5	107.1	108.4	99.95	115.38
	HOP-92	120.9	109.3	107.7	110.12	115.4
	NCI-H226	99.82	94.57	91.94	96.96	100.71
	NCI-H23	102.4	93.59	93.98	100.32	97.03
	NCI-H322M	94	96.1	98.49	101.21	95.89
	NCI-H460	116.7	121.1	107.8	106.44	118.73
	NCI-H522	96.78	94.82	96.41	97.3	96.7
*Colon cancer*	COLO 205	111.6	111.5	108.9	100.1	113.79
	HCC-2998	105.2	107.6	104.5	101.52	104.81
	HCT-116	97.82	100.6	97.06	104.92	110.15
	HCT-15	99.39	94.99	94.12	101.91	100.49
	HT29	103.4	109.3	107.1	104.06	113.18
	KM12	100.2	99.16	98.57	99.5	100.71
	SW-620	103.7	108.3	99.5	96.91	101.88
*CNS cancer*	SF-268	97.06	97.16	96.17	94.28	104.11
	SF-295	94.85	98.09	99.52	96.86	101.54
	SF-539	95.37	92.59	92.35	94.08	95.62
	SNB-19	97.85	96.23	96.31	98.24	97.73
	U251	103.1	107.2	105.5	104.47	104.72
*Melanoma*	LOX IMVI	96.84	87.19	84.52	92.19	96.42
	MALME-3M	93.65	94.22	96.35	94.53	94.69
	M14	101.2	101.9	101.8	101.76	104.17
	MDA-MB-435	97.22	96.66	96.81	100.53	99.08
	SK-MEL-28	99.56	102.6	104.4	104.47	101.02
	SK-MEL-5	92.01	86.59	90.69	92.19	93.38
	UACC-257	102	105.8	104.4	94.53	103.72
	UACC-62	90.53	85.6	80.81	101.76	93.41
*Ovarian cancer*	IGROV1	105	106.3	104.3	100.53	110.36
	OVCAR-3	109.2	113.9	111.8	104.47	106.37
	OVCAR-4	97.78	96.67	93.93	94.18	99.61
	OVCAR-5	104.8	102.9	101.1	105.8	102.63
	OVCAR-8	99.87	103.1	102.2	92.98	97.77
	NCI/ADR-RES	102.4	102.1	103.1	108.87	102.13
	SK-OV-3	102.6	105.6	99.28	107.11	104.8
*Renal cancer*	786-0	98.32	100.2	100.1	100.59	104.72
	A498	103.9	117	114.4	108.88	115.67
	ACHN	98.77	92.38	89.61	99.89	99.4
	CAKI-1	92.91	82.3	78.52	99.75	89.58
	RXF 393	96.91	102	105.8	114.5	105.46
	SN 12C	93.94	93.88	94.56	93.07	99.81
	TK-10	96.11	109.1	113.5	102.14	111.49
*Prostate cancer*	PC-3	99.99	102.2	99.48	96.67	104.34
	DU-145	104.9	104.6	103.3	105.71	104.85
*Breast cancer*	MCF7	84.87	90.11	83.48	87.43	91.63
	HS 578T	98.14	96.97	92.76	93.19	100.05
	BT-549	103.5	117.7	112.2	117.25	115.87
	T-47D	103.8	102.3	98.97	103.41	105.35
	MDA-MB-468	99.41	97.99	100	98.35	97.8
Mean GP		99.92	100.11	98.79	100.64	102.35

**Table 3:** The anticancer activity (GP and PGI) of the five most sensitive cell lines at 10 µM.

Compound	Anticancer Activity in One Dose (10 µM)		
	The Most Sensitive Cell Lines	GP	PGI
**7a**	MCF7 (Breast cancer)	84.87	15.13
	HL-60(TB) (Leukemia)	86.23	13.77
	EKVX (Non-small cell lung cancer)	89.44	10.56
	SK-MEL-5 (Melanoma)	92.01	7.99
	CAKI-1 (Renal cancer)	92.91	7.09
**7b**	EKVX (Non-small cell lung cancer)	80.73	19.27
	CAKI-1 (Renal cancer)	82.3	17.7
	HL-60(TB) (Leukemia)	83.07	16.93
	UACC-62 (Melanoma)	85.6	14.4
	LOX IMVI (Melanoma)	87.19	12.81
**7c**	EKVX (Non-small cell lung cancer)	75.46	24.54
	CAKI-1 (Renal cancer)	78.52	21.48
	UACC-62 (Melanoma)	80.81	19.19
	MCF7 (Breast cancer)	83.48	16.52
	LOX IMVI (Melanoma)	84.52	15.48
**7e**	MCF7 (Breast cancer)	87.43	12.57
	EKVX (Non-small cell lung cancer)	90.83	9.17
	LOX IMVI (Melanoma)	92.19	7.81
	SK-MEL-5 (Melanoma)	92.19	7.81
	SR (Leukemia)	92.66	7.34
**7f**	CAKI-1 (Renal cancer)	89.58	10.42
	EKVX (Non-small cell lung cancer)	91.2	8.8
	MCF7 (Breast cancer)	91.63	8.37
	K-562 (Leukemia)	92.54	7.46
	SR (Leukemia)	93.35	6.47

**Table 4:** The average PGI of the 1-(1,3-dioxoisoindolin-2-yl)-3-aryl urea analogs (**7a**–**f**) and imatinib at 10 µM.

Panels	7a	7b	7c	7e	7f	Imatinib *	Gefitinib *	Thalidomide *
*Leukemia*	1.76	2.72	1.82	−2.6	1.27	9	**79.68**	15.28
*Non-small cell lung cancer*	−2.94	0.01	2.07	−0.7	−3.85	15.68	**63.97**	0.06
*Colon cancer*	−3.05	−4.5	−1.39	−1.3	−6.43	5.34	**52.19**	−0.27
*CNS cancer*	2.36	1.74	2.02	2.41	−0.74	5.8	**46.13**	−4.95
*Melanoma*	3.38	4.93	5.03	2.26	1.76	−0.87	**44.99**	−0.39
*Ovarian cancer*	−3.08	−4.38	−2.23	−1.99	−3.38	−7.16	**60.93**	1.90
*Renal cancer*	2.74	0.45	0.50	−2.69	−3.733	3.25	**77.89**	−2.37
*Prostate cancer*	−2.46	−3.40	−1.41	−1.19	−4.59	12.5	**59.60**	−8.60
*Breast cancer*	2.06	−0.99	2.25	0.07	−2.35	12.15	**52.88**	−2.04

The best outcome is indicated by bold font; * The anticancer data of gefitinib and thalidomide with NSC Codes 759854 and 66847, respectively, were retrieved from official website of NCI, USA [44].

**Table 5:** The molecular docking studies of 1-(1,3-dioxoisoindolin-2-yl)-3-aryl urea analogs (**7a**–**f**).

S. No.	Compound	PDB: 1SA0		PDB ID: 1SC7		PDB ID: 3W2R		
		Docking Score	Emodel Score	Docking Score	Emodel Score	Docking Score	Emodel Score	Interaction
1	**7a**	−6.469	−54.331	−7.169	−59.500	−7.934	−66.771	H-bond (Lys745; 2.05 Å), π-π-stacking (Phe856; 5.21 Å)
2	**7b**	−6.426	−56.002	−7.473	−63.130	−7.816	−64.552	H-bond (Lys745; 1.97 Å)
3	**7c**	−6.480	−57.825	−7.318	−64.802	−7.558	−64.096	H-bond (Lys745; 2.04 Å)
4	**7d**	−7.069	−57.606	−7.565	−66.006	−7.702	−65.761	H-bond (Lys745; 2.04 Å), π-π-stacking (Phe856; 5.48 Å)
5	**7e**	−7.292	−62.624	−6.363	−65.436	−7.119	−65.715	H-bond (Lys745; 1.64 Å), H-bond (Asp855; 2.13 Å)
6	**7f**	−5.830	−54.813	−7.306	−63.183	−8.644	−65.599	H-bond (Thr854 via H_2_O; 1.76 and 2.39 Å)

**Table 6:** The DPPH free radical scavenging activity of compounds **7a**–**f**.

S. No.	Compound	Free Radical Scavenging Activity IC_50_ (μM)
1	**7a**	36.18 ± 0.31
2	**7b**	39.22 ± 0.12
3	**7c**	24.02 ± 0.29
4	**7d**	16.05 ± 0.15
5	**7e**	54.27 ± 0.49
6	**7f**	15.99 ± 0.10
7	Ascorbic acid	14.02 ± 0.15

**Table 7:** In silico ADME prediction of 1-(1,3-dioxoisoindolin-2-yl)-3-aryl urea analogs (**7a**–**f**).

S. No.	Compound	% ABS	TPSA	HBA (<10)	HBD (<5)	NROTB	Log *p* (<5)	MW (500)	GI Absorption	Lipinski’s Violation (≤1)
1	**7a**	81.91	78.51	4	2	4	1.74	299.26	High	0
2	**7b**	81.51	78.51	3	2	4	1.92	315.71	High	0
3	**7c**	81.51	78.51	3	2	4	2.03	360.16	High	0
4	**7d**	78.73	87.74	4	2	5	1.93	311.29	High	0
5	**7e**	81.51	78.51	3	2	4	1.94	315.71	High	0
6	**7f**	78.73	87.74	4	2	5	2.05	311.29	High	0

% ABS = Percent absorption; TPSA = Total polar surface area; HBAs = Hydrogen bond acceptors; HBDs = Hydrogen bond donors; NROTB = Number of rotatable bonds; Log *p* = Lipophilicity; MW = Molecular weight; GI = Gastrointestinal.

**Table 8:** In silico toxicity prediction of 1-(1,3-dioxoisoindolin-2-yl)-3-aryl urea analogs (**7a**–**f**).

S. No.	Compound	HEP	IMMUNO	MUTAGEN	CYTOTOX	LD_50_ (mg/Kg)	Toxicity Class
1	**7a**	+	−	−	−	1000	IV
2	**7b**	+	−	−	−	5000	V
3	**7c**	+	−	−	−	1000	IV
4	**7d**	+	−	−	−	5000	V
5	**7e**	+	−	−	−	1500	IV
6	**7f**	+	−	−	−	5000	V

HEP = Hepatotoxicity; IMMUNO = Immunotoxicity; MUTAGEN = Mutagenicity; CYTOTOX = Cytotoxicity; + = active; − = inactive.

## Data Availability

The Supplementary Material to this article contains the information that supports the findings of this study.

## Supplementary Materials

The following supporting information can be downloaded at: https://www.mdpi.com/article/10.3390/molecules29010067/s1, Figures S1–S5: anticancer data of compound **7a**–**c**, **7e**,**f** against 56 cancer cell lines at 10 µM; Figures S6–S16: characterization data of compounds **7a**–**f**. Table S1: the molecular docking studies of 1-(1,3-dioxoisoindolin-2-yl)-3-aryl urea analogs (**7a**–**f**).
