# Supplementary material for: An Efficient Synthesis of 1-(1,3-Dioxoisoindolin-2-yl)-3-aryl Urea Analogs as Anticancer and Antioxidant Agents: An Insight into Experimental and In Silico Studies
Source: Molecules. 2023 Dec 21;29(1):67. doi: 10.3390/molecules29010067 (PMC10779787; doi:10.3390/molecules29010067)
Supplement: Supplementary file 1 [file molecules-29-00067-s001.zip › molecules-2700296-supplementary.pdf]

An Efficient Synthesis of 1-(1,3-Dioxoisoindolin-2-yl)-3-Aryl Urea Analogs as Anticancer and Antioxidant Agents: An Insight into the Experimental and *In Silico* Studies

Obaid Afzal [1,*], and Mohamed Jawed Ahsan [2]

[1] Department of Pharmaceutical Chemistry, College of Pharmacy, Prince Sattam Bin Abdulaziz University, Al-Kharj 11942, Saudi Arabia
[2] Department of Pharmaceutical Chemistry, Faculty of Pharmacy, Jahangirabad Institute of Technology (JIT), Jahangirabad Fort, Jahangirabad, Barabanki Uttar Pradesh 225 203 India; jawedpharma@gmail.com
* Correspondence: o.akram@psau.edu.sa or obaid263@gmail.com

SUPPLEMENTARY MATERIALS

Materials and Methods

Chemistry

All the chemicals were of synthetic grades. The melting point were recorded by open capillary method and progress of reaction analyzed by thin layer chromatography plate (TLC Silica gel 60 F254). The nuclear magnetic resonance (NMR) and mass spectra were recorded on a Bruker AC 300 MHz spectrometer and waters ACQUITY TQD respectively.

Molecular docking studies

Table S1. The molecular docking studies of 1-(1,3-dioxoisoindolin-2-yl)-3-aryl urea analogs (**7a-f**).

S. No.	Compound	PDB: 1SA0	PDB ID: 1SC7
		Electrostatic interaction	Electrostatic interaction
1	**7a**	Lys425 (π-Cationic; 4.88 Å)	–
2	**7b**	–	–
3	**7c**	–	–
4	**7d**	Ala317 (H-bond; 2.69 Å); Lys352 (π-Cation; 6.00 Å)	Tyr426 (H-bond; 1.83 Å); Met428 (H-bond; 2.08)
5	**7e**	Asn258 (H-bond; 2.28 Å); Lys254 (π-Cation; 3.91 Å); Val181 (Halogen bond; 2.95 Å)	Tyr426 (H-bond; 1.18 Å); Met428 (H-bond; 2.05)
6	**7f**	Lys254 (π-Cation; 3.57 Å)	Arg364 (H-bond; 2.15 Å)

(−) No significant electrostatic interaction was observed in docking studies; * Unspecified residue

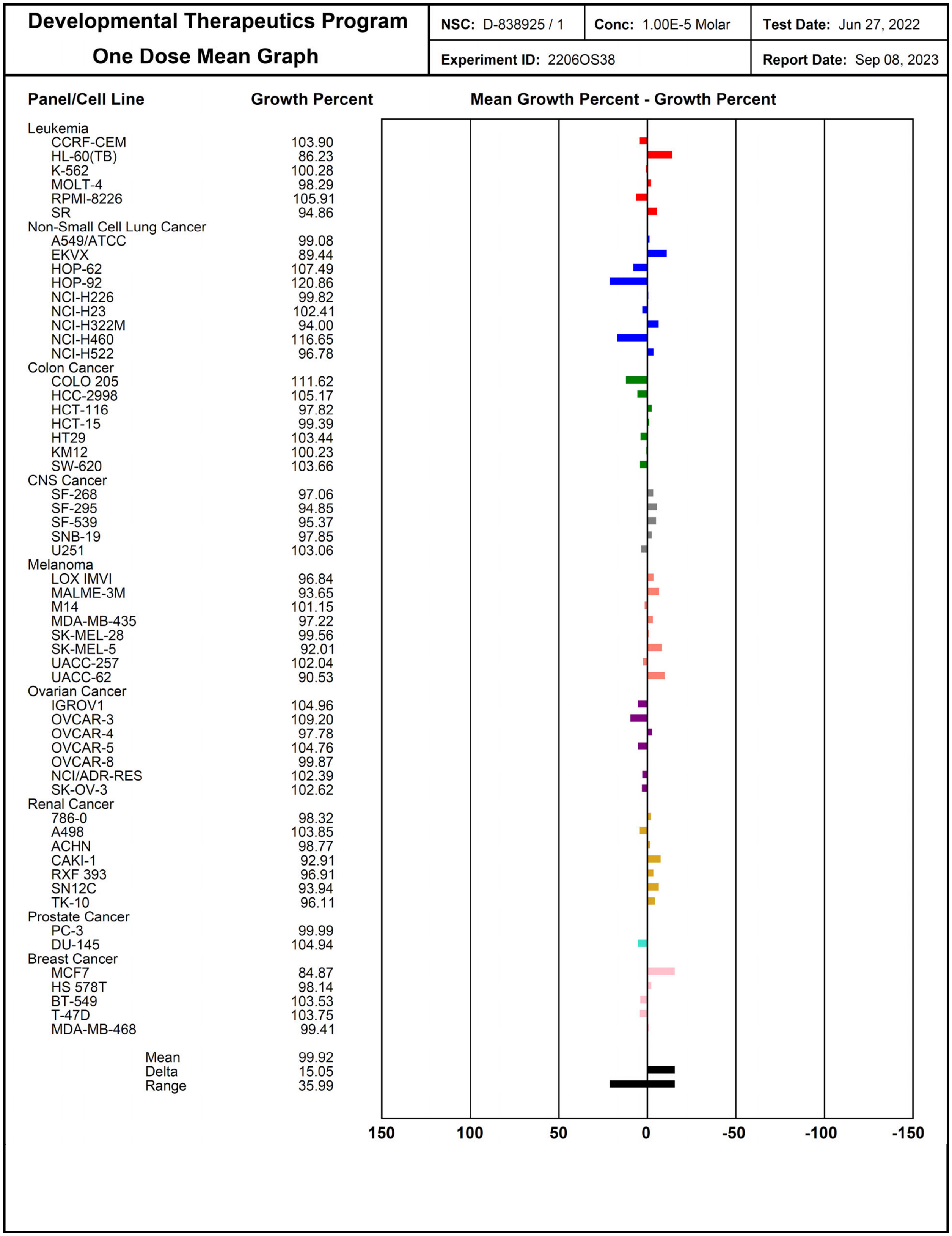

Figure S1. Anticancer data of compound **7a** against 56 cancer cell lines at 10 μM.

Developmental Therapeutics Program

One Dose Mean Graph

NSC: D-838926 / 1 | **Conc:** 1.00E-5 Molar | **Test Date:** Jun 27, 2022

Experiment ID: 2206OS38 | **Report Date:** Sep 08, 2023

Panel/Cell Line	Growth Percent
Leukemia	
CCRF-CEM	104.81
HL-60(TB)	83.07
K-562	91.98
MOLT-4	101.13
RPMI-8226	106.47
SR	96.20
Non-Small Cell Lung Cancer	
A549/ATCC	102.67
EKVX	80.73
HOP-62	107.09
HOP-92	109.30
NCI-H226	94.57
NCI-H23	93.59
NCI-H322M	96.10
NCI-H460	121.06
NCI-H522	94.82
Colon Cancer	
COLO 205	111.47
HCC-2998	107.56
HCT-116	100.56
HCT-15	94.99
HT29	109.30
KM12	99.16
SW-620	108.30
CNS Cancer	
SF-268	97.16
SF-295	98.09
SF-539	92.59
SNB-19	96.23
U251	107.21
Melanoma	
LOX IMVI	87.19
MALME-3M	94.22
M14	101.92
MDA-MB-435	96.66
SK-MEL-28	102.62
SK-MEL-5	86.59
UACC-257	105.76
UACC-62	85.60
Ovarian Cancer	
IGROV1	106.31
OVCAR-3	113.91
OVCAR-4	96.67
OVCAR-5	102.88
OVCAR-8	103.14
NCI/ADR-RES	102.14
SK-OV-3	105.62
Renal Cancer	
786-0	100.22
A498	117.00
ACHN	92.38
CAKI-1	82.30
RXF 393	101.98
SN12C	93.88
TK-10	109.10
Prostate Cancer	
PC-3	102.24
DU-145	104.57
Breast Cancer	
MCF7	90.11
HS 578T	96.97
BT-549	117.65
T-47D	102.27
MDA-MB-468	97.99
Mean	100.11
Delta	19.38
Range	40.33

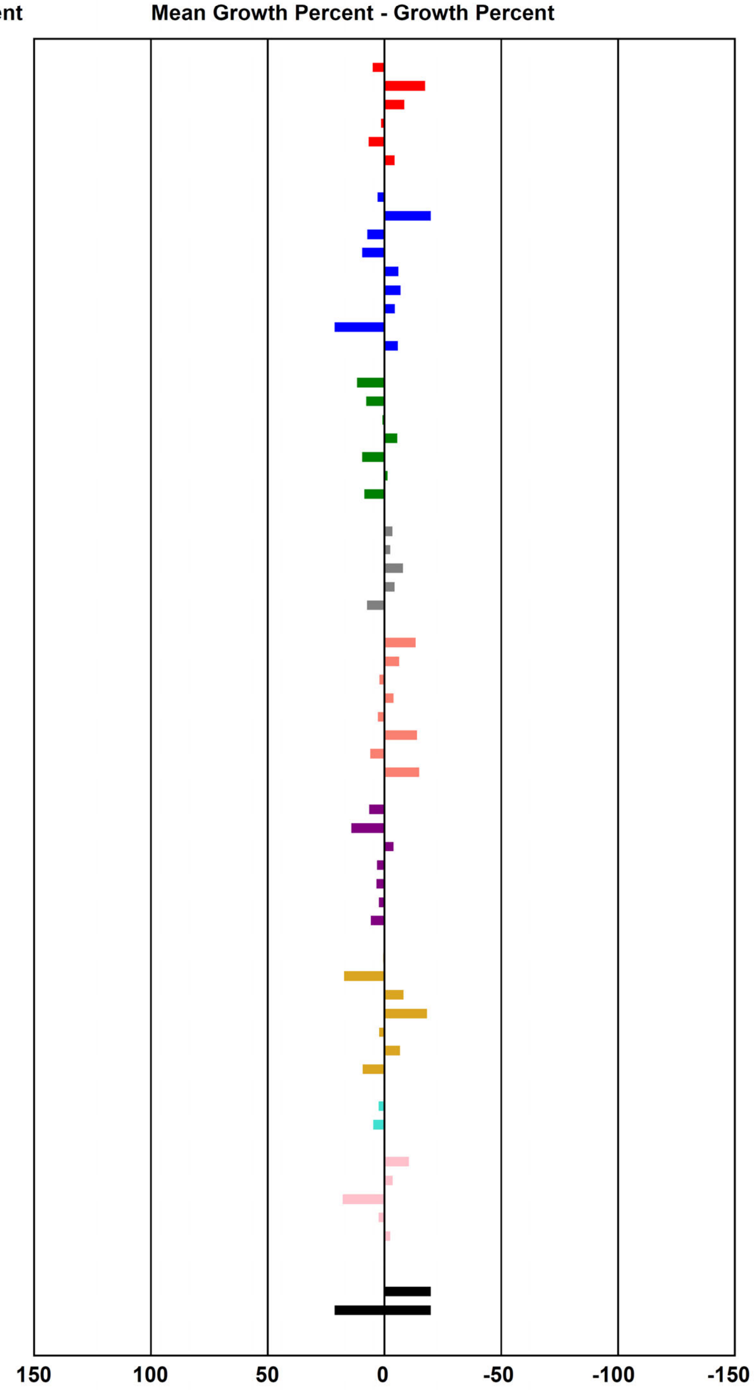

Figure S2. Anticancer data of compound **7b** against 56 cancer cell lines at 10 μM.

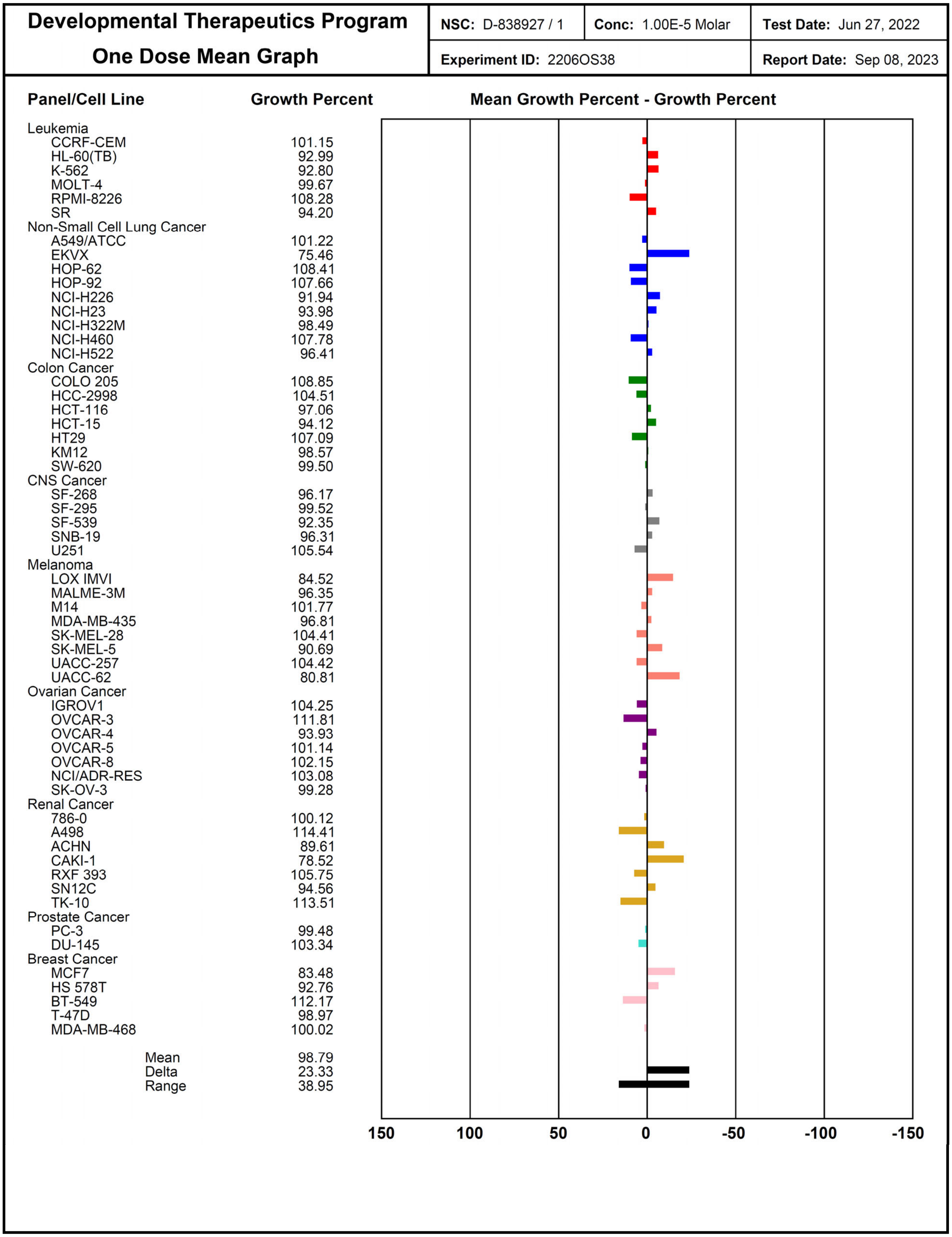

Figure S3. Anticancer data of compound **7c** against 56 cancer cell lines at 10 μM.

Developmental Therapeutics Program

One Dose Mean Graph

NSC: D-838928 / 1	Conc: 1.00E-5 Molar	Test Date: Jun 27, 2022
Experiment ID: 2206OS38		Report Date: Sep 08, 2023

Panel/Cell Line	Growth Percent
Leukemia	
CCRF-CEM	103.37
HL-60(TB)	101.97
K-562	110.70
MOLT-4	97.49
RPMI-8226	109.46
SR	92.66
Non-Small Cell Lung Cancer	
A549/ATCC	103.02
EKVX	90.83
HOP-62	99.95
HOP-92	110.12
NCI-H226	96.96
NCI-H23	100.32
NCI-H322M	101.21
NCI-H460	106.44
NCI-H522	97.30
Colon Cancer	
COLO 205	100.10
HCC-2998	101.52
HCT-116	104.92
HCT-15	101.91
HT29	104.06
KM12	99.50
SW-620	96.91
CNS Cancer	
SF-268	94.28
SF-295	96.86
SF-539	94.08
SNB-19	98.24
U251	104.47
Melanoma	
LOX IMVI	92.19
MALME-3M	94.53
M14	101.76
MDA-MB-435	100.53
SK-MEL-28	104.47
SK-MEL-5	94.18
UACC-257	105.80
UACC-62	92.98
Ovarian Cancer	
IGROV1	108.87
OVCAR-3	107.11
OVCAR-4	100.59
OVCAR-5	108.88
OVCAR-8	99.89
NCI/ADR-RES	99.75
SK-OV-3	96.46
Renal Cancer	
786-0	105.89
A498	106.14
ACHN	97.95
CAKI-1	87.71
RXF 393	114.50
SN12C	93.07
TK-10	102.14
Prostate Cancer	
PC-3	96.67
DU-145	105.71
Breast Cancer	
MCF7	87.43
HS 578T	93.19
BT-549	117.25
T-47D	103.41
MDA-MB-468	98.35
Mean	100.64
Delta	13.21
Range	29.82

Mean Growth Percent - Growth Percent

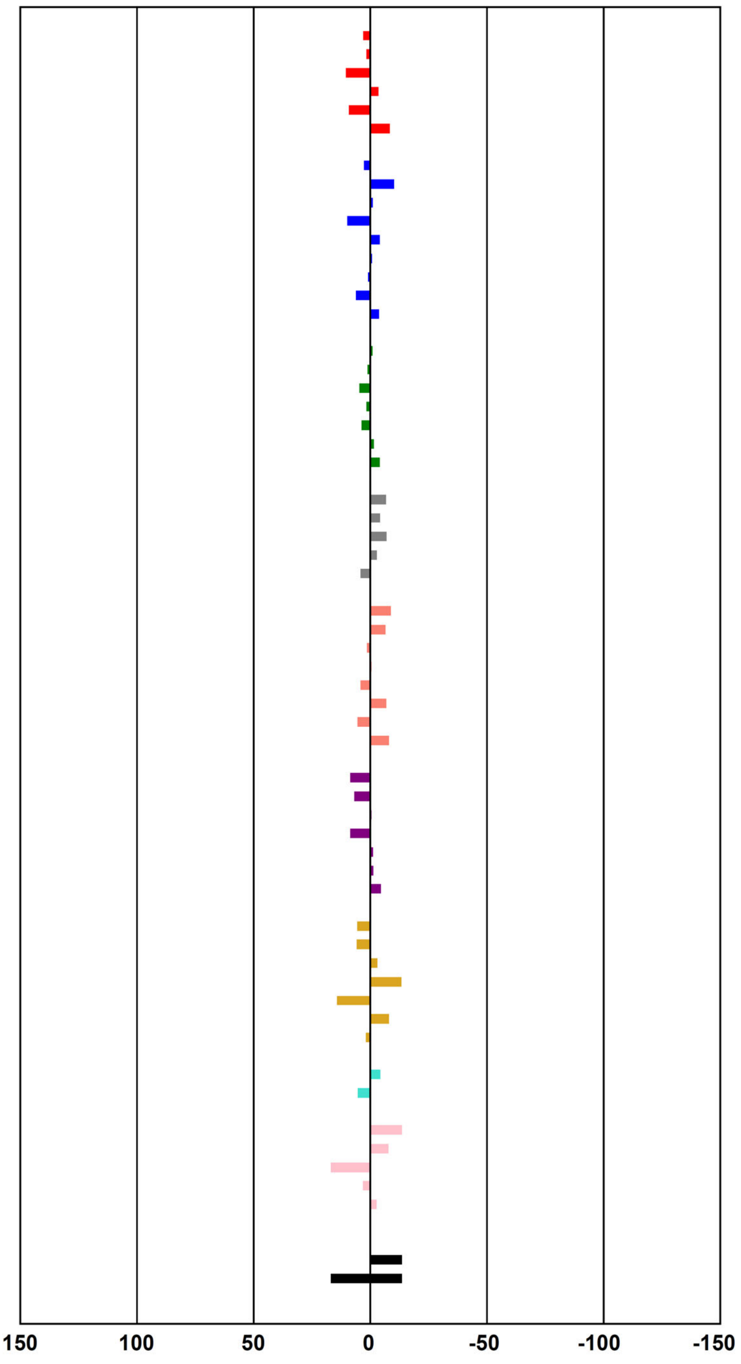

Figure S4. Anticancer data of compound **7e** against 56 cancer cell lines at 10 μM.

Developmental Therapeutics Program

One Dose Mean Graph

**NSC:** D-838934 / 1	**Conc:** 1.00E-5 Molar	**Test Date:** Jun 27, 2022
**Experiment ID:** 2206OS38		**Report Date:** Sep 08, 2023

Panel/Cell Line	Growth Percent
Leukemia	
CCRF-CEM	95.13
HL-60(TB)	101.22
K-562	92.54
MOLT-4	106.20
RPMI-8226	103.93
SR	93.35
Non-Small Cell Lung Cancer	
A549/ATCC	103.59
EKVX	91.20
HOP-62	115.38
HOP-92	115.40
NCI-H226	100.71
NCI-H23	97.03
NCI-H322M	95.89
NCI-H460	118.73
NCI-H522	96.70
Colon Cancer	
COLO 205	113.79
HCC-2998	104.81
HCT-116	110.15
HCT-15	100.49
HT29	113.18
KM12	100.71
SW-620	101.88
CNS Cancer	
SF-268	104.11
SF-295	101.54
SF-539	95.62
SNB-19	97.73
U251	104.72
Melanoma	
LOX IMVI	96.42
MALME-3M	94.69
M14	104.17
MDA-MB-435	99.08
SK-MEL-28	101.02
SK-MEL-5	93.38
UACC-257	103.72
UACC-62	93.41
Ovarian Cancer	
IGROV1	110.36
OVCAR-3	106.37
OVCAR-4	99.61
OVCAR-5	102.63
OVCAR-8	97.77
NCI/ADR-RES	102.13
SK-OV-3	104.80
Renal Cancer	
786-0	104.72
A498	115.67
ACHN	99.40
CAKI-1	89.58
RXF 393	105.46
SN12C	99.81
TK-10	111.49
Prostate Cancer	
PC-3	104.34
DU-145	104.85
Breast Cancer	
MCF7	91.63
HS 578T	100.05
BT-549	115.87
T-47D	105.35
MDA-MB-468	97.80
Mean	102.35
Delta	12.77
Range	29.15

Mean Growth Percent - Growth Percent

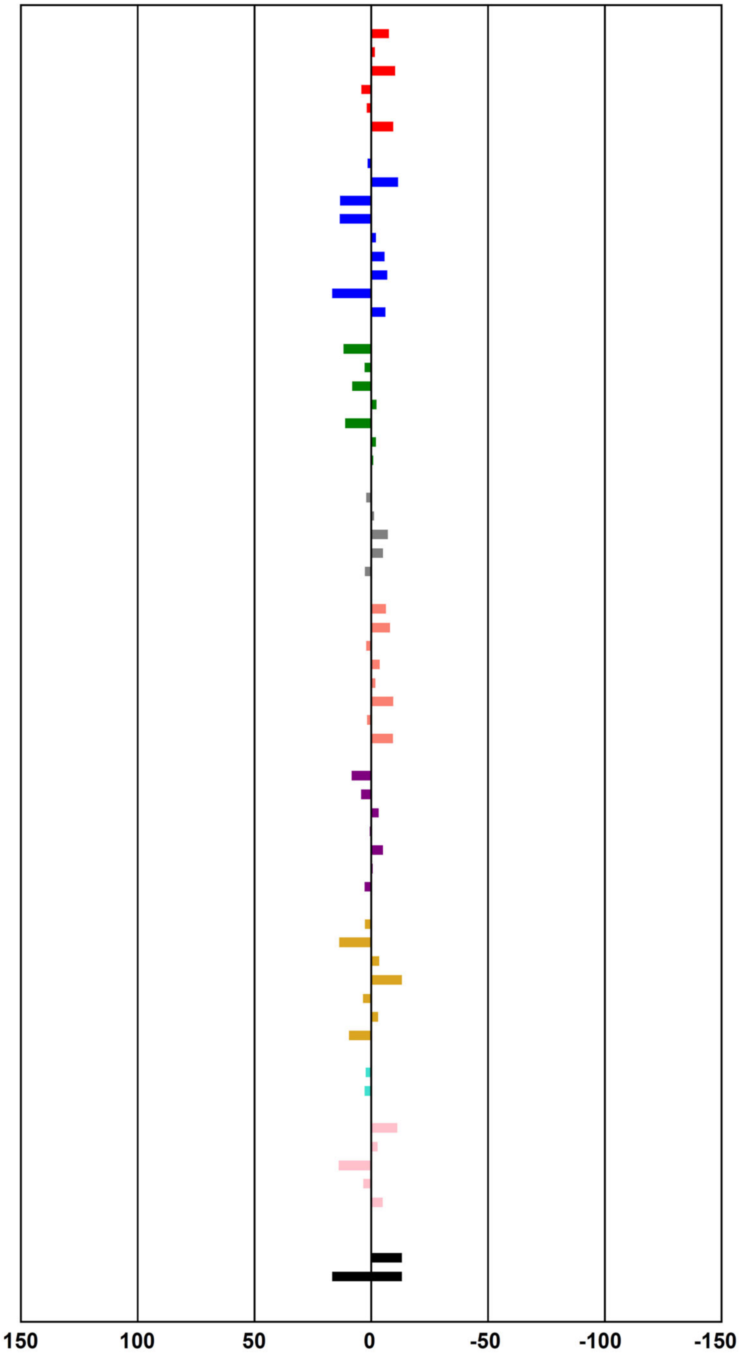

Figure S5. Anticancer data of compound **7f** against 56 cancer cell lines at 10 μM.

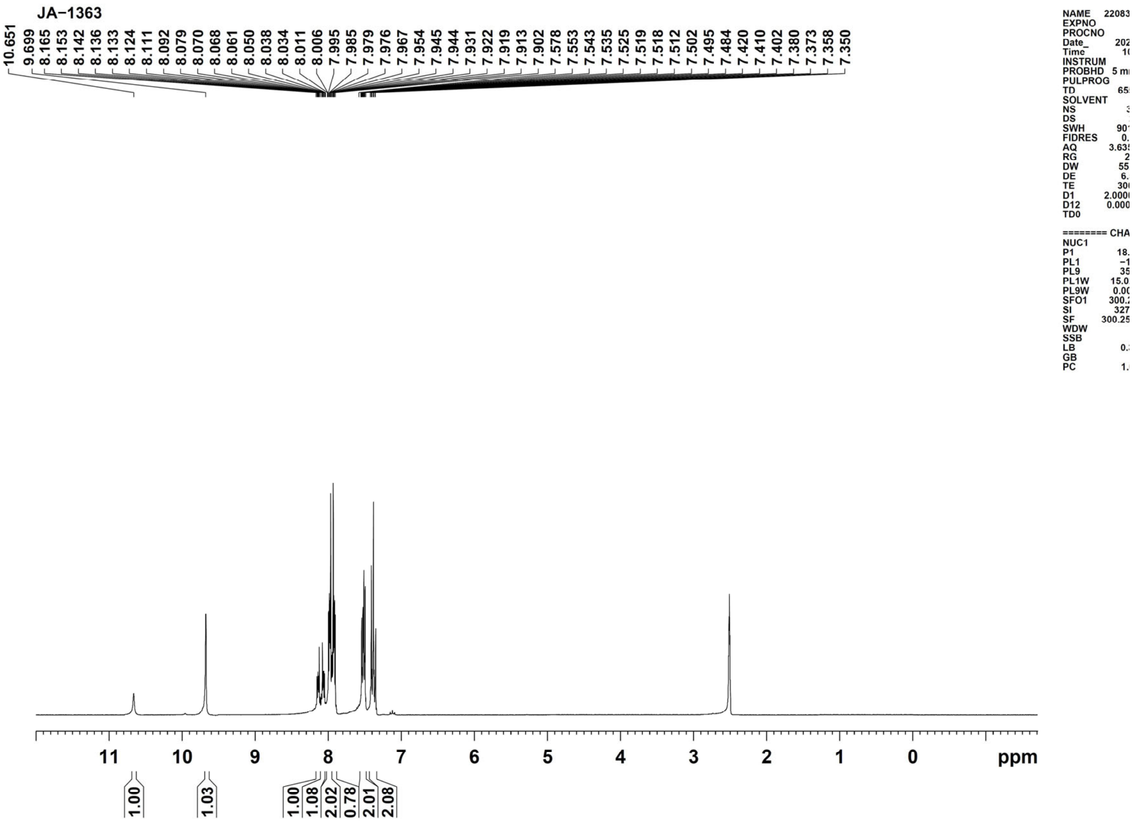

Figure S6. ^{1}H NMR spectra of the compound **7a**.

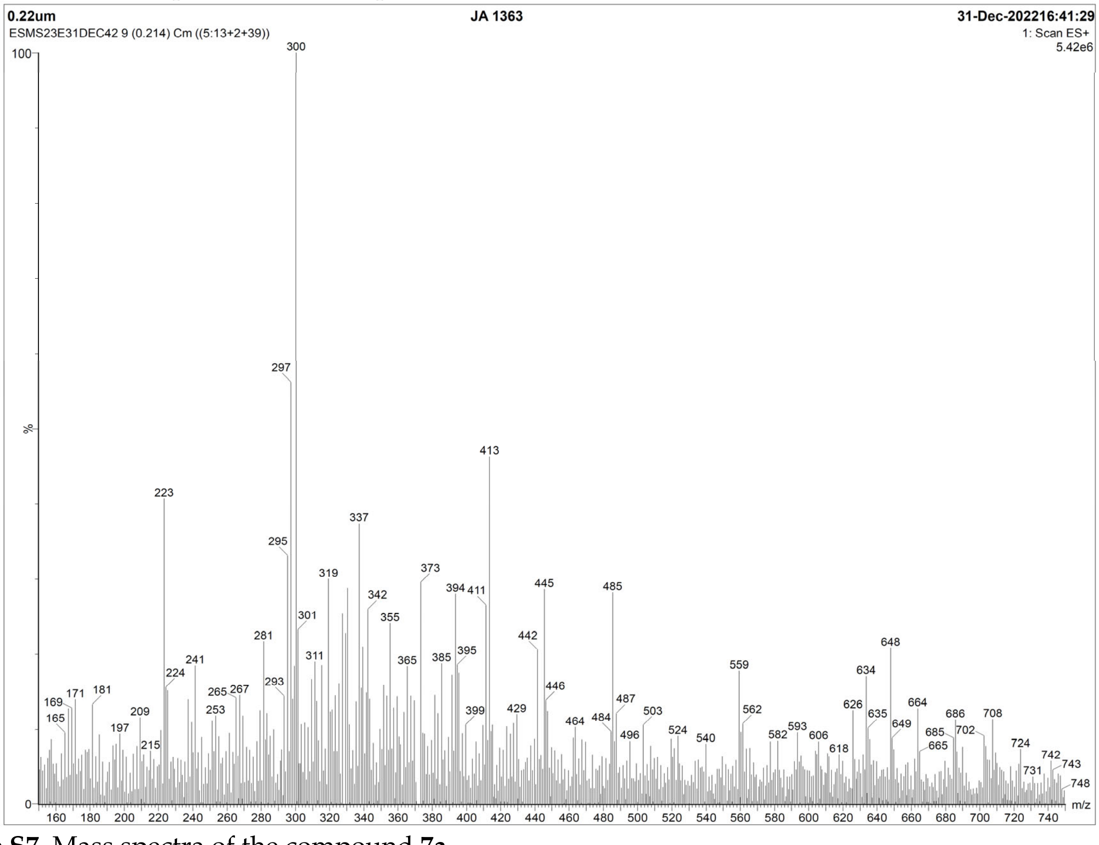

Figure S7. Mass spectra of the compound **7a**.

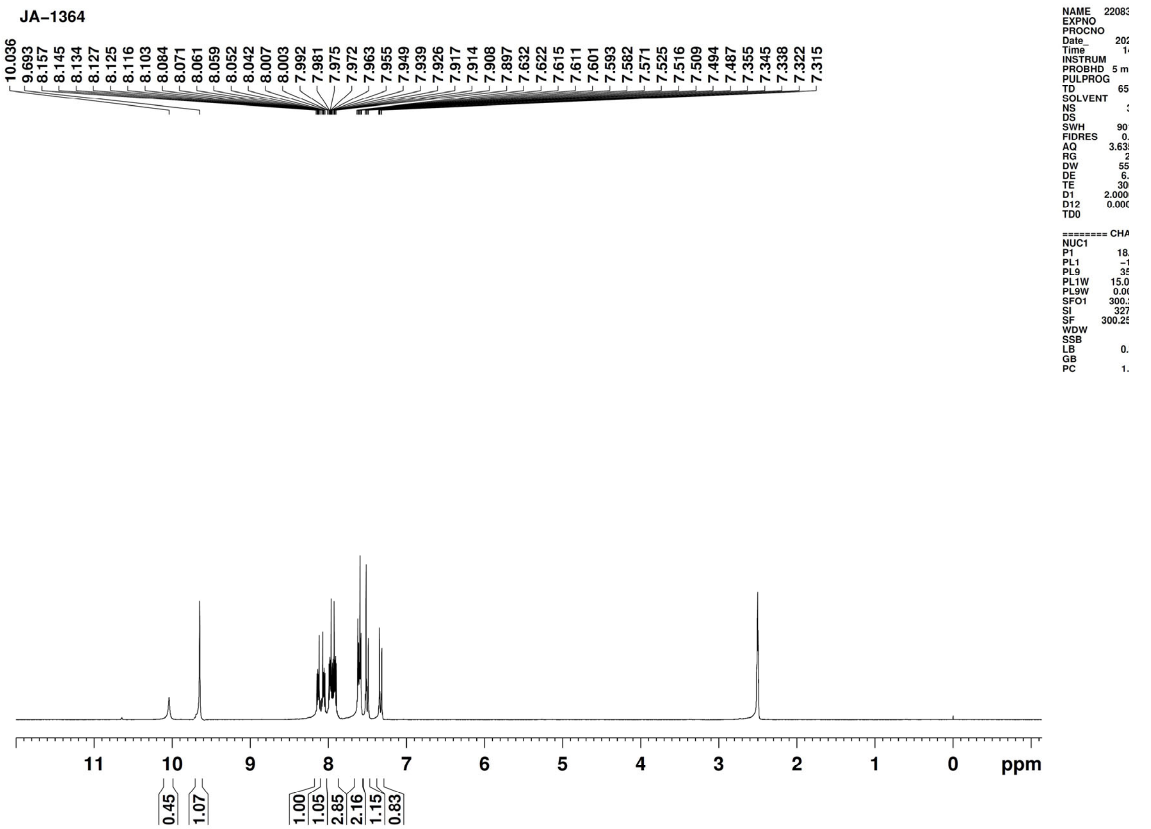

Figure S8. ^{1}H NMR spectra of the compound **7b**.

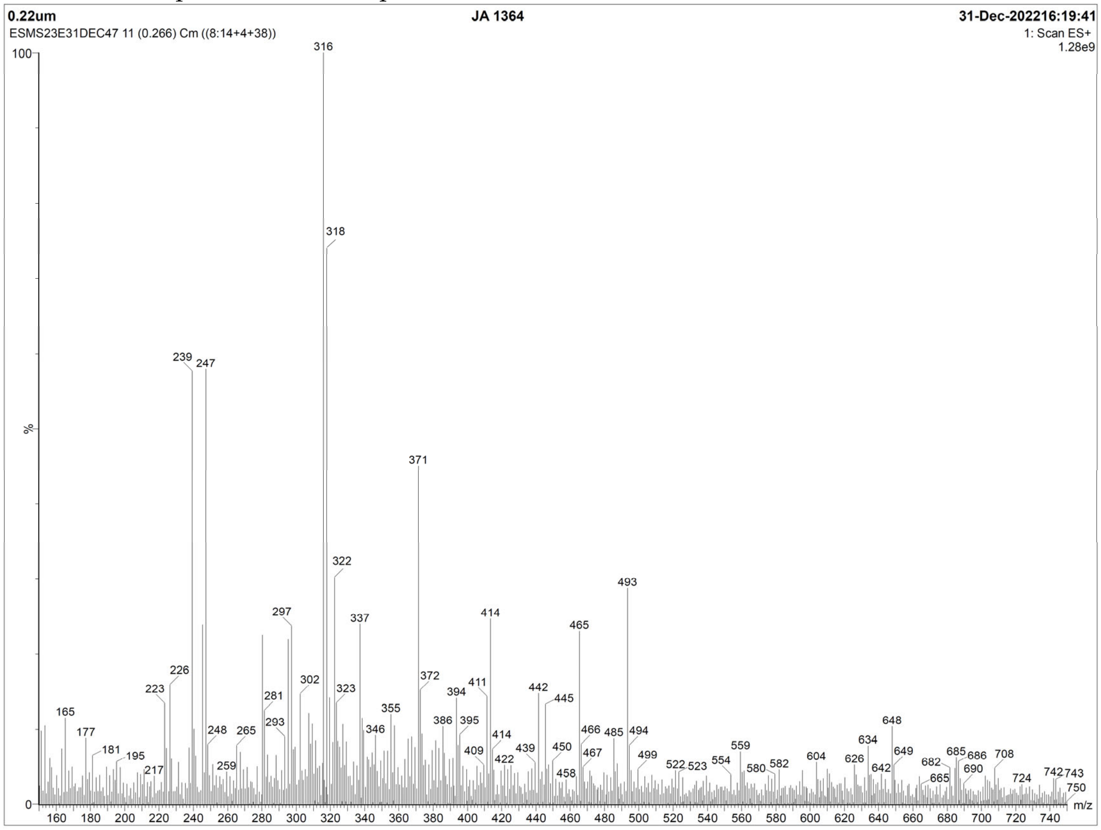

Figure S9. Mass spectra of the compound **7b**.

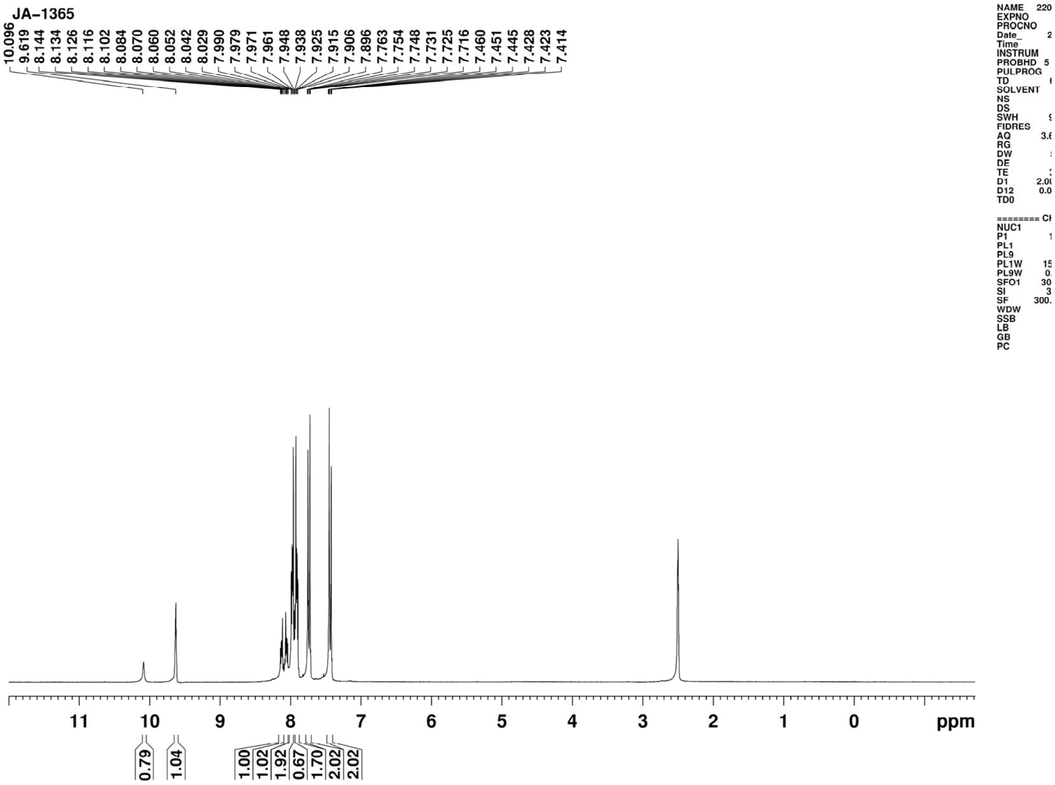

Figure S10. ^{1}H NMR spectra of the compound **7c**.

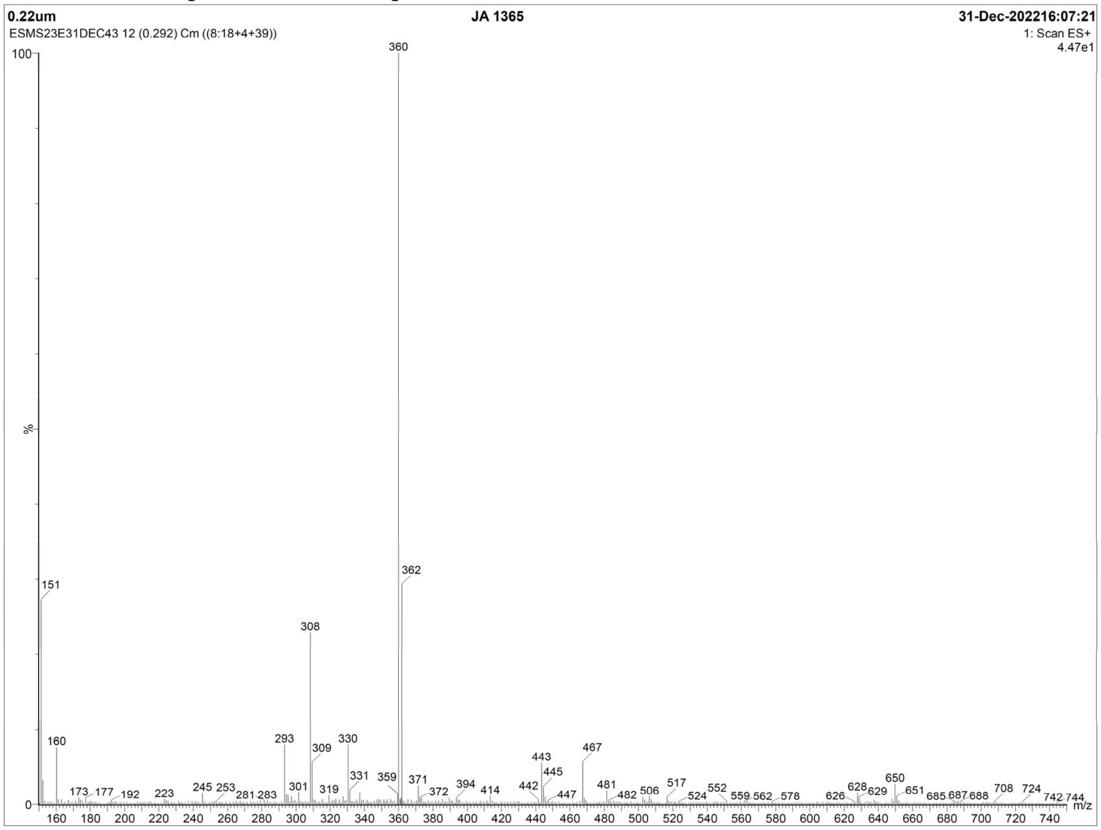

Figure S11. Mass spectra of the compound **7c**.

HPLC GRAPH

Analyzer: Mr.Bhawani
Spl.Name: Unknown Sample-02
Wave.L:210 nm
Batch No:02
Status:
Flow:1.000 ml/min
Column: C18
Col.Temp:25
Date:2023-07-27 15:44:14

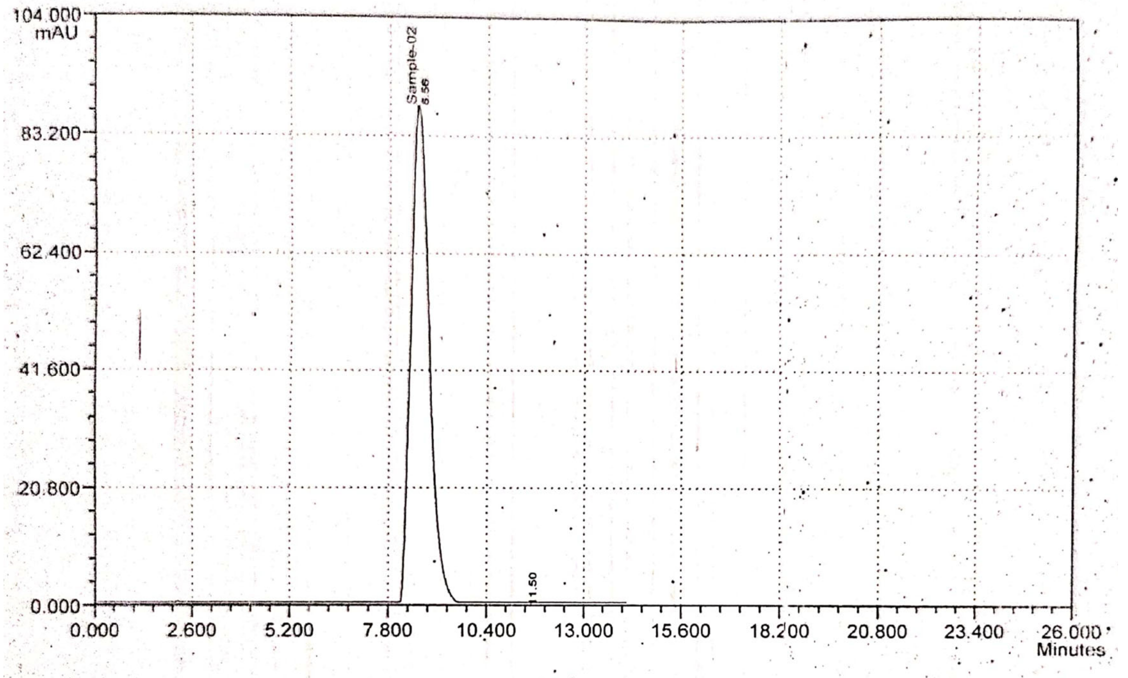

ID	Name	Retain.T	Height	Area	Conc	Tail.Factor	Theo.Plate
1	Sample-02	8.557	8953	312594.6	98.743	2.03	1197
2		11.499	128	3978.0	1.257	1.05	2728
	Sum:		9081	316572.6	100.0000		

Figure S12. HPLC chromatogram of the compound **7c**.

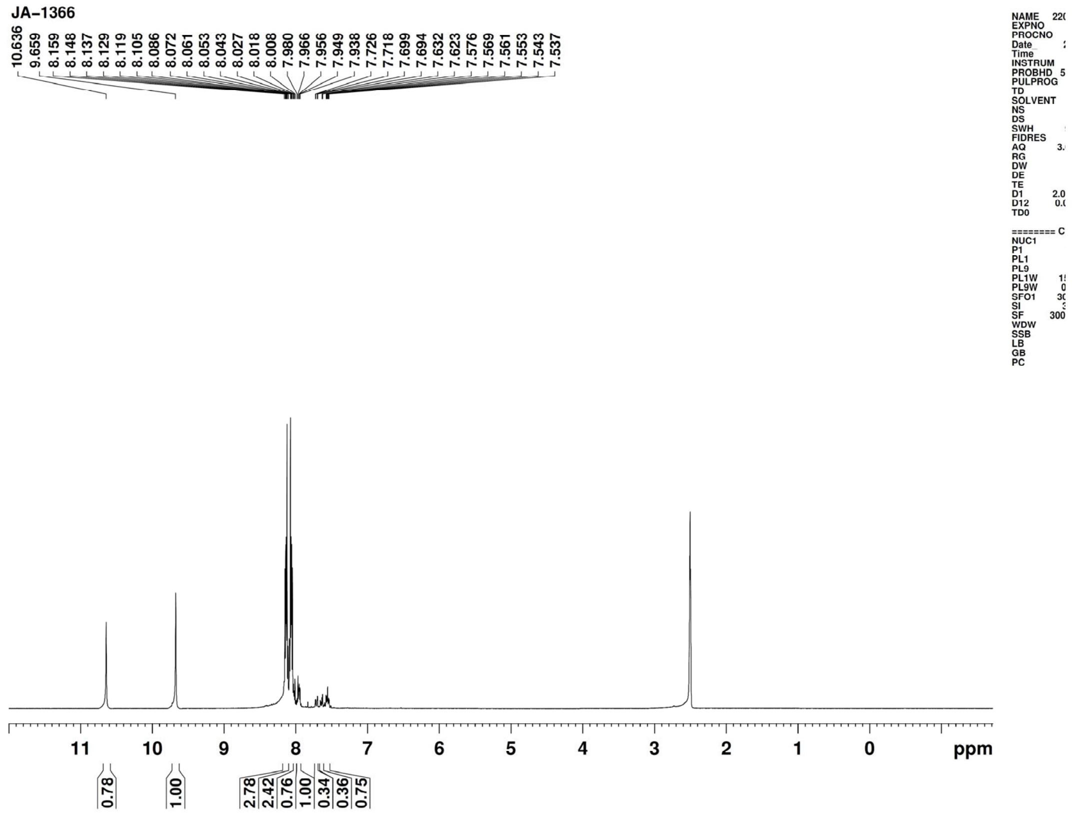

Figure S13. ^{1}H NMR spectra of the compound **7e**.

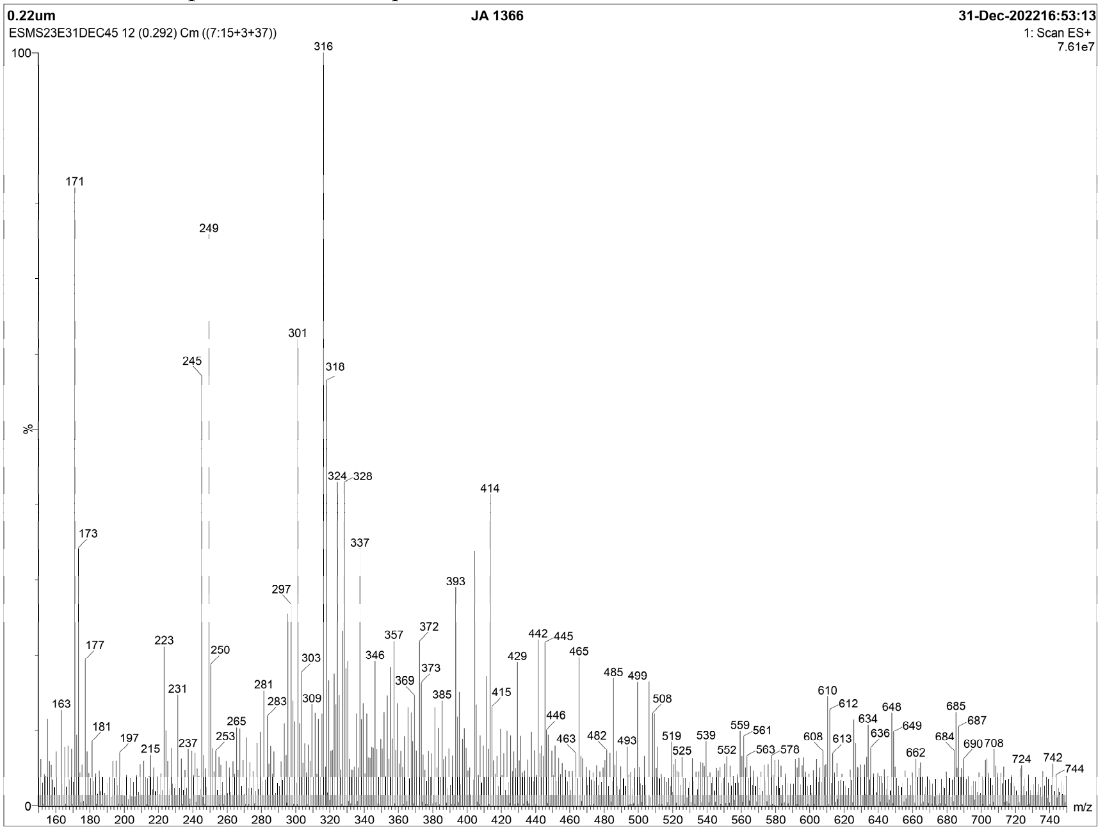

Figure S14. Mass spectra of the compound **7e**.

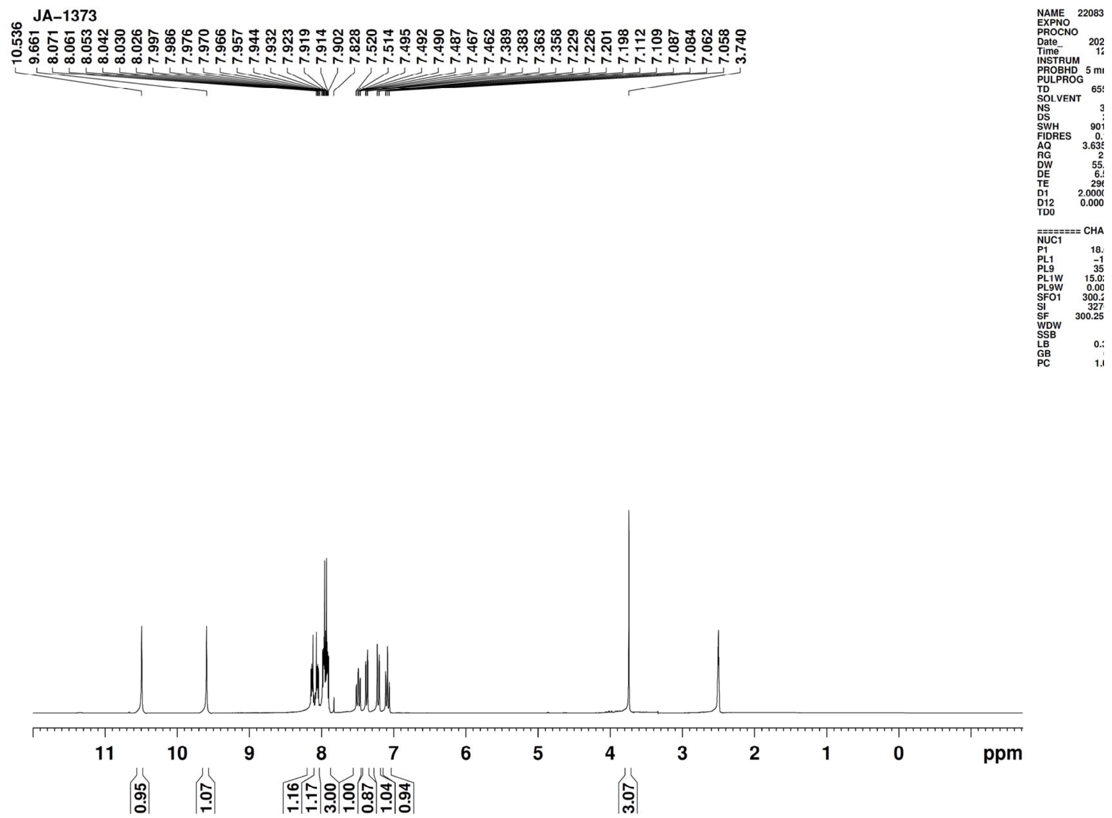

Figure S15. ^{1}H NMR spectra of the compound **7f**.

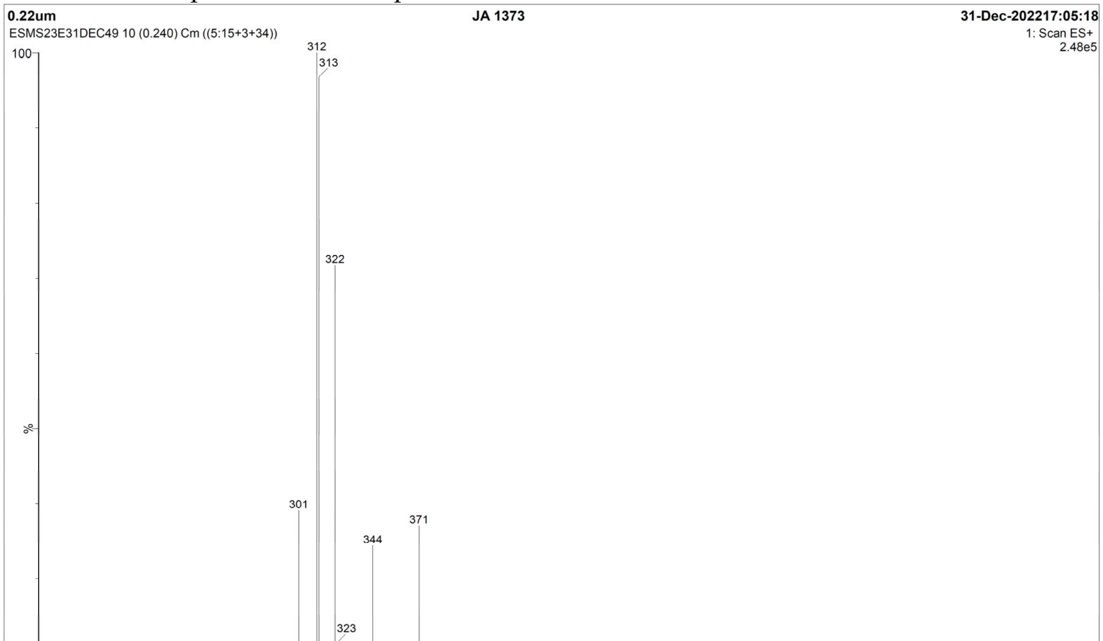

Figure S16. Mass spectra of the compound **7f**.
